# A review on photocatalytic materials: synthesis strategies, morphology, and mechanisms

**DOI:** 10.1039/d6na00157b

**Published:** 2026-09-09

**Authors:** Smita Korpal, Mohamed A. Habib, Jasvir Dalal, Pounraj Thanasekaran, Pawan Kumar, Supreet, Supriya Sehrawat, Ahmed B. M. Ibrahim

**Affiliations:** a Department of Physics, Rajdhani College, University of Delhi Delhi-110015 India jasvirdalal2012@gmail.com jasvir@rajdhani.du.ac.in; b Department of Chemistry, College of Science, Imam Mohammad Ibn Saud Islamic University (IMSIU) Riyadh 11623 Saudi Arabia; c Department of Chemistry, Rajdhani College, University of Delhi Delhi 110015 India; d Department of Chemistry, Pondicherry University Puducherry – 605 014 India; e Department of Physics, Amity School of Applied Sciences, Amity University Haryana 122413 India; f Department of Physics, Deenbandhu Chhotu Ram University of Science and Technology Sonipat 131039 India

## Abstract

Photocatalysis is a promising approach for solar energy conversion and environmental remediation. Despite extensive research, advances in photocatalytic materials remain limited. Many reported performance improvements rely on empirical optimization rather than a clear mechanistic understanding. Existing reviews mainly focus on material classification and performance. However, they rarely examine how synthesis strategies control structure, defects, and charge-transfer behavior in a reproducible manner. This concept-driven critical review addresses these limitations by examining how synthesis methods influence structural evolution and charge carrier dynamics in photocatalytic materials. Rather than summarizing material types and applications, the review focuses on the role of crystallinity, defect chemistry, morphology, and interfacial properties in determining photocatalytic performance. Advanced architectures, including hierarchical structures and Z- & S-scheme heterojunctions, are assessed to understand the actual origin of their enhanced photocatalytic performance. In addition, the review highlights important issues related to mechanistic validation and inconsistent performance reporting that are often overlooked in conventional reviews. This review integrates synthesis strategies, charge-transfer mechanisms, and performance evaluation approaches to provide practical strategies for designing efficient, stable, and scalable photocatalytic materials for environmental and energy-related applications.

## Abstract 2

Photocatalysis is a promising approach for solar energy conversion and environmental remediation.

## Graphical abstract

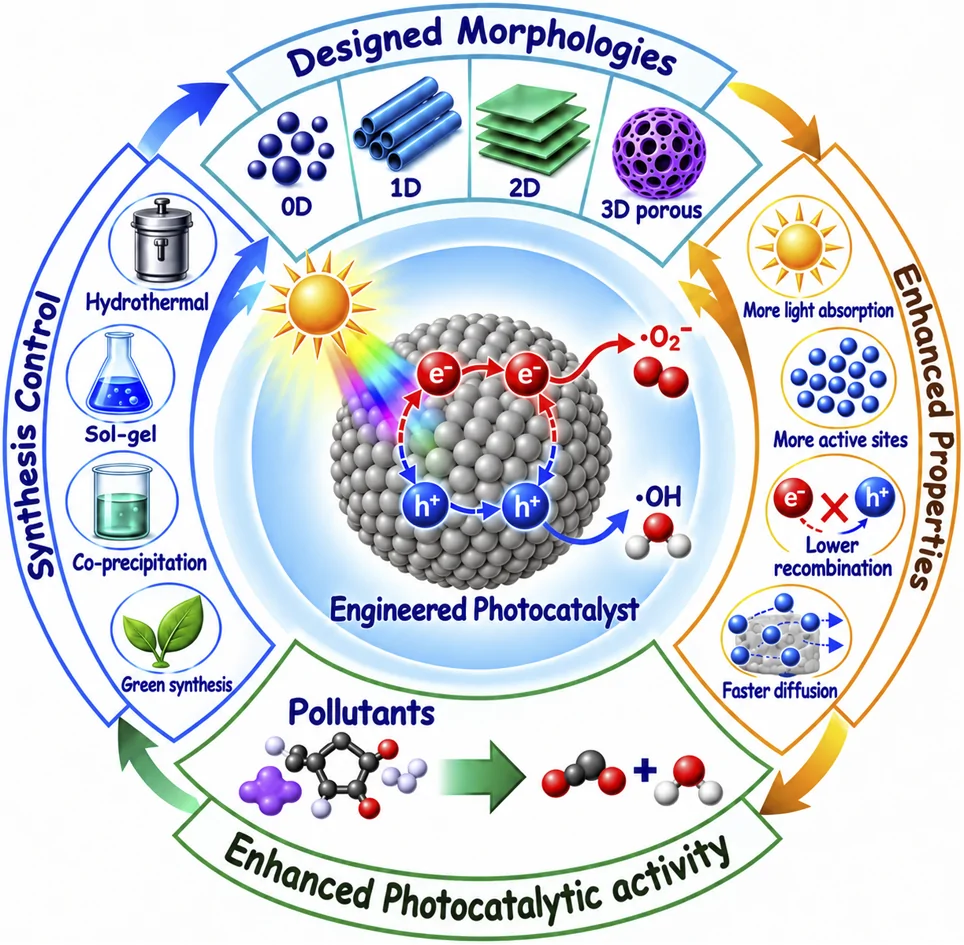

## 1 Introduction

The growing demand for clean energy and the urgent need to address environmental pollution have made photocatalysis an important area of research. By utilizing light to activate semiconductor materials, photocatalytic systems can drive redox reactions^1–4^ relevant to H_2_ evolution, CO_2_ conversion, pollutant degradation, and water disinfection.^5–8^ These broad applications have attracted great interest in developing photocatalysts with improved efficiency, stability, and visible-light activity. Thus, the research activity in photocatalysis has increased significantly in recent years as depicted in Fig. 1. This trend reflects the growing interest in developing efficient and sustainable photocatalytic materials.^9^ It also highlights the increasing demand for advanced synthesis strategies that can precisely control the structural and functional properties of photocatalysts.

**Fig. 1:**
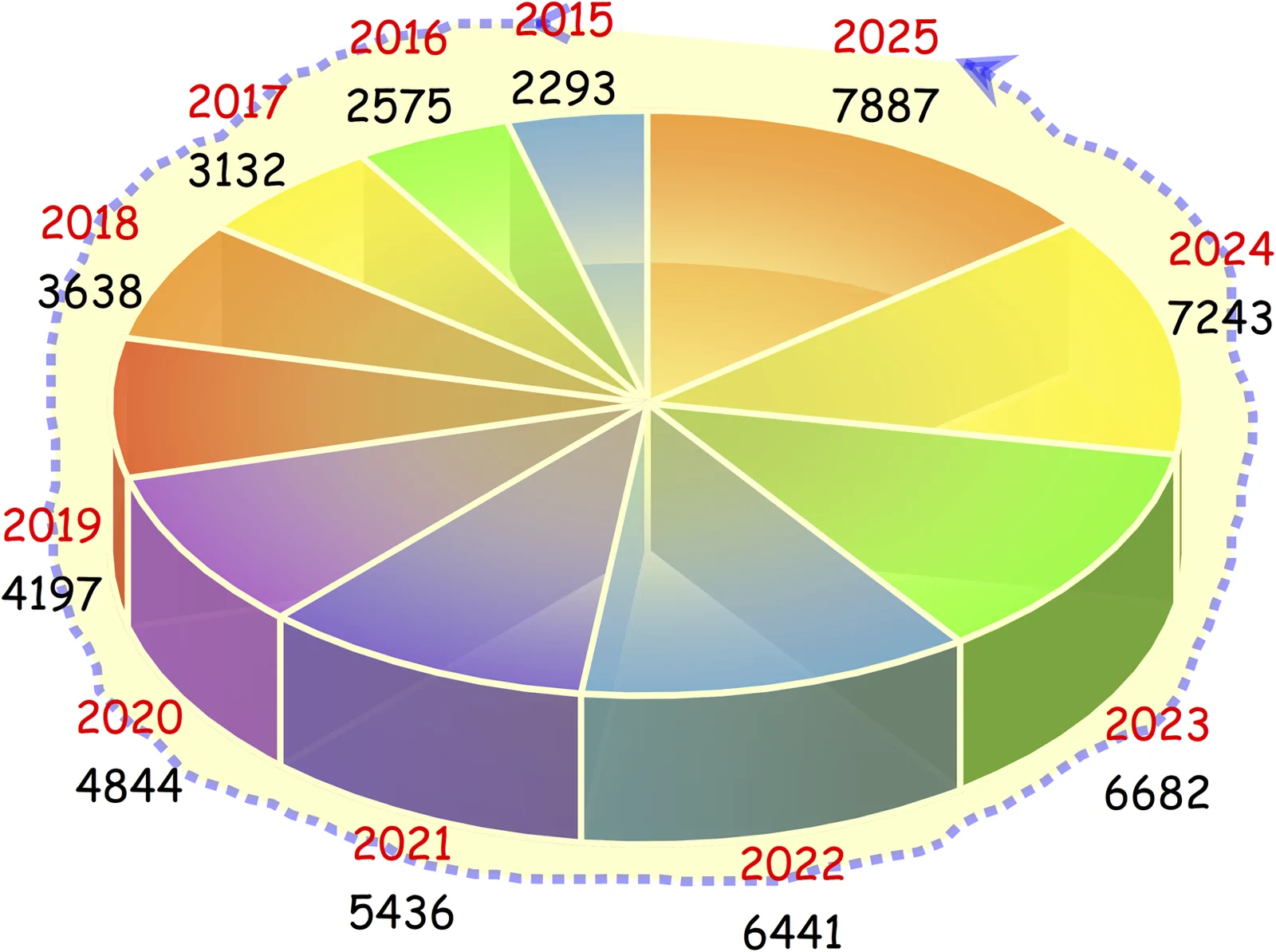
Emerging research trends in the field of photocatalysis based on publication data retrieved from the Scopus database (Keywords: photocatalysis, photocatalytic activity, photocatalysts, photodegradation).

Among the many factors that influence photocatalytic performance, synthesis route, morphology, defect chemistry, and interfacial structure are especially important. Conventional materials such as TiO_2_ and ZnO remain widely studied because of their stability and favorable redox properties, but their wide band gaps limit solar utilization.^10–12^ To overcome this limitation, researchers have explored doping, heterostructure construction, defect engineering,^13,14^ and morphology control across a wide range of material families, including metal oxides,^15^ chalcogenides,^16,17^ MOFs,^18–20^ carbon-based systems,^21–23^ and hybrid composites. Morphological design has been extensively used to improve photocatalytic behavior. Zero-dimensional nanostructures can increase surface-active sites,^24^ one-dimensional architectures can support directional charge transport,^25,26^ two-dimensional layers can promote interfacial charge transfer,^27^ and three-dimensional hierarchical structures can improve light harvesting and mass transport.^28,29^ Also, heterostructures such as core–shell systems, and Z- & S-scheme junctions are often reported to suppress charge recombination and enhance redox performance^30–32^ Defect engineering has also emerged as an effective strategy for tuning the electronic structure of photocatalysts.^33^ The introduction of oxygen vacancies, dopants, and surface functional groups can improve visible-light absorption and create active sites for photocatalytic reactions.^34,35^ These defects can enhance charge separation and reactant adsorption; however, excessive defects may act as recombination centers and reduce material stability. Therefore, advanced synthesis approaches such as atomic layer deposition, plasma treatment, and post-synthetic modification are increasingly being used to achieve controlled defect engineering.

Recent studies show that synthesis conditions, defect engineering, and heterointerface design can strongly affect charge-carrier lifetime, apparent quantum efficiency (AQE), H_2_ evolution rate, and long-term stability. The ALD-grown TiO_2_ films showed an increase in carrier lifetime from a few picoseconds in amorphous samples to more than 400 ps after crystallization at 500 °C, highlighting the importance of structural ordering.^36^ Moreover, the Zn-vacancy-mediated Z-scheme CdS/ZnS heterojunctions exhibited much higher H_2_ evolution activity because of improved interfacial charge transfer,^37^ whereas the S-scheme ZnCdS/ZnS systems also showed enhanced hydrogen production and charge separation due to vacancy engineering. In addition, MnCdS/ZnS-VZn heterostructures achieved an AQE of 16.43% at 420 nm together with excellent photostability,^38^ indicating simultaneous improvement in efficiency and durability. Thus, the above literature provides a more reliable indication for mechanistic improvement than isolated photocatalytic activity values alone through different morpho-structures.

Existing reviews mainly discuss material types, morphology, or heterostructure design separately, with limited focus on how synthesis methods reproducibly control the structural features that influence charge-carrier dynamics. This review addresses this gap by analyzing how synthesis strategies control structural growth. It further examines how these structural changes influence charge separation, charge transport, and photocatalytic performance, thereby distinguishing it from earlier reviews that consider morphology and heterostructures mainly as design categories. The novelty of this review lies in correlating synthesis strategies, morphostructural control, defect chemistry, and mechanistic validation within a single critical framework. Thus, by sharpening the connection between synthesis, structure, mechanism, and evaluation, this review aims to identify realistic design principles for developing robust and scalable photocatalysts for energy and environmental applications.

## 2 Scope of the review

This review critically examines how synthesis strategies influence the morphostructure, defect chemistry, and interfacial properties of photocatalysts, and how these factors affect charge separation, charge transport, and photocatalytic performance. Instead of only classifying materials based on type or application, the review focuses on whether the reported structure and performance correlation is supported and reproducible. The review covers major photocatalyst families, including metal oxides, chalcogenides, carbon-based materials, polymeric semiconductors, metal–organic frameworks, and hybrid heterostructures. The literature was selected based on four main criteria: relevance to synthesis-controlled structural evolution, discussion of morphology, inclusion of mechanistic evidence related to charge-carrier behavior, and sufficient experimental details for critical comparison. To improve transparency, studies focused only on nominal activity enhancement without structural or mechanistic validation were not emphasized. Similarly, reports lacking sufficient experimental details or relying only on indirect evidence for charge transfer were evaluated with caution. Highly application-specific studies with limited general design relevance were also not extensively discussed. To improve transparency, studies focused only on activity enhancement without proper structural or mechanistic validation were not considered.

This review differs from many earlier critical reviews by organizing the discussion around synthesis-induced structural evolution rather than only material categories or heterostructure types. Morphology, defect engineering, and heterojunction design are discussed together within a common framework of synthesis control, mechanistic validation, and performance evaluation. This approach helps identify which photocatalyst design strategies are scientifically reliable and potentially scalable for practical applications.

## 3 Important factors for photocatalysis

Photocatalytic performance depends on several interconnected factors rather than a single structural feature as shown in Fig. 2. The synthesis route, crystallinity, defect density, morphology, and interfacial structure together influence light absorption, charge generation, charge separation, migration, and surface reaction kinetics.^39,40^ Therefore, improvements in photocatalytic activity should not be attributed only to morphology or composition without clear experimental validation and reliable performance analysis. Morphology is often considered a direct indicator of photocatalytic efficiency, but reported results are more complex. Zero-dimensional structures can provide more active sites, one-dimensional architectures can improve charge transport, two-dimensional sheets can enhance interfacial transfer, and three-dimensional hierarchical structures can improve light harvesting and mass transport.^40,41^ However, these advantages are not universal. In reported TiO_2_-based systems, it is observed that a 0D/1D composite showed better performance than pure 0D particles despite having a lower surface area, suggesting that reduced recombination and improved phase interaction may be more important than surface area alone.^42^ Thus, a higher surface area does not promise better photocatalytic activity. Defect engineering also shows both positive and negative effects. Vacancies, dopants, and surface defects can improve visible-light absorption and enhance charge separation.^43,44^ However, excessive defects may act as recombination centers and reduce structural stability. Therefore, defect concentration should be optimized. Heterostructure design is another widely used strategy, especially in Z- & S-scheme systems.^45,46^

**Fig. 2:**
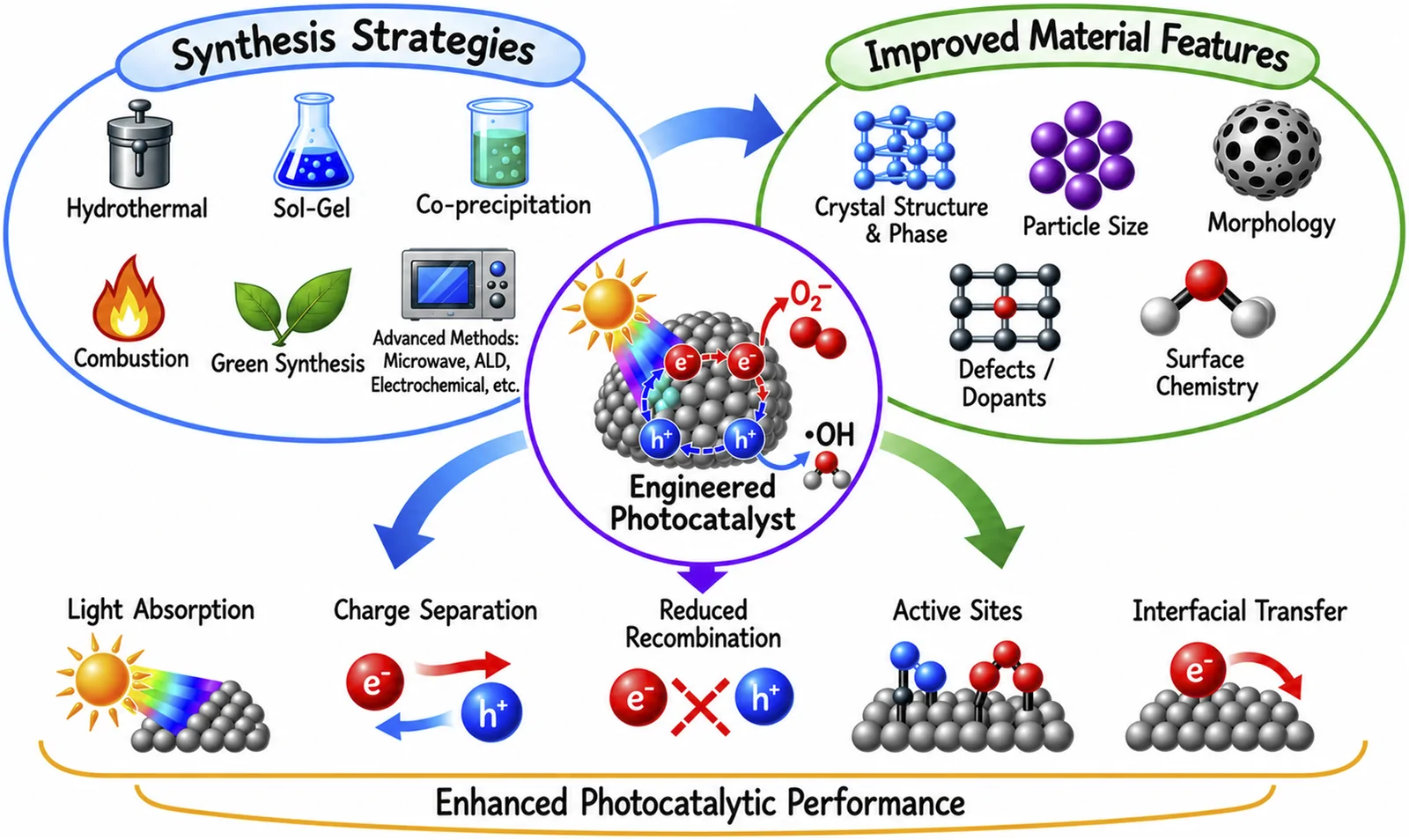
Schematic illustration of the correlation between synthesis strategies and photocatalytic performance.

Photocatalytic efficiency is significantly influenced by the dynamics of photogenerated charge carriers, including their generation, separation, migration, trapping, transfer, and recombination processes.^47^ Upon light irradiation with energy equal to or greater than the semiconductor band gap, electrons are excited from the valence band to the conduction band, leaving positively charged holes in the VB. The subsequent fate of these charge carriers determines the overall photocatalytic activity because rapid electron–hole recombination significantly reduces quantum efficiency and limits surface redox reactions.^39^ The migration and separation behavior of charge carriers are strongly influenced by crystallinity, defect states, morphology, exposed crystal facets, and interfacial electric fields. Highly crystalline structures generally exhibit lower recombination because of reduced trap-assisted scattering, while controlled defect engineering can introduce shallow trapping states that prolong carrier lifetime and enhance visible-light absorption.^48^ In contrast, excessive defects may create deep trap states that accelerate non-radiative recombination. Morphological engineering also contributes significantly to charge dynamics. Thus, efficient examination of charge carrier dynamics is essential for achieving high photocatalytic efficiency.

## 4 Synthesis methods for photocatalysts

Various researchers have found that the physicochemical properties and photocatalytic action of any photocatalyst are highly varied due to its preparation method. The common photocatalyst preparation methods include co-precipitation, sol–gel, sonochemical, hydrothermal, solvothermal, chemical vapour deposition, *etc.* as shown in Fig. 3. Furthermore, Table 1 summarizes photocatalytic materials synthesized using different fabrication methods and their photocatalytic applications. The reported studies show that the synthesis strategy strongly affects the morphostructure, defect chemistry, charge separation, and light absorption behavior. Conventional methods such as hydrothermal, sol–gel, co-precipitation, and microwave-assisted synthesis remain widely used because of their simplicity and good control over crystallinity and morphology. In addition, heterostructure engineering and Z-scheme design significantly improve visible-light activity, charge transfer, stability, and photocatalytic efficiency.

**Fig. 3:**
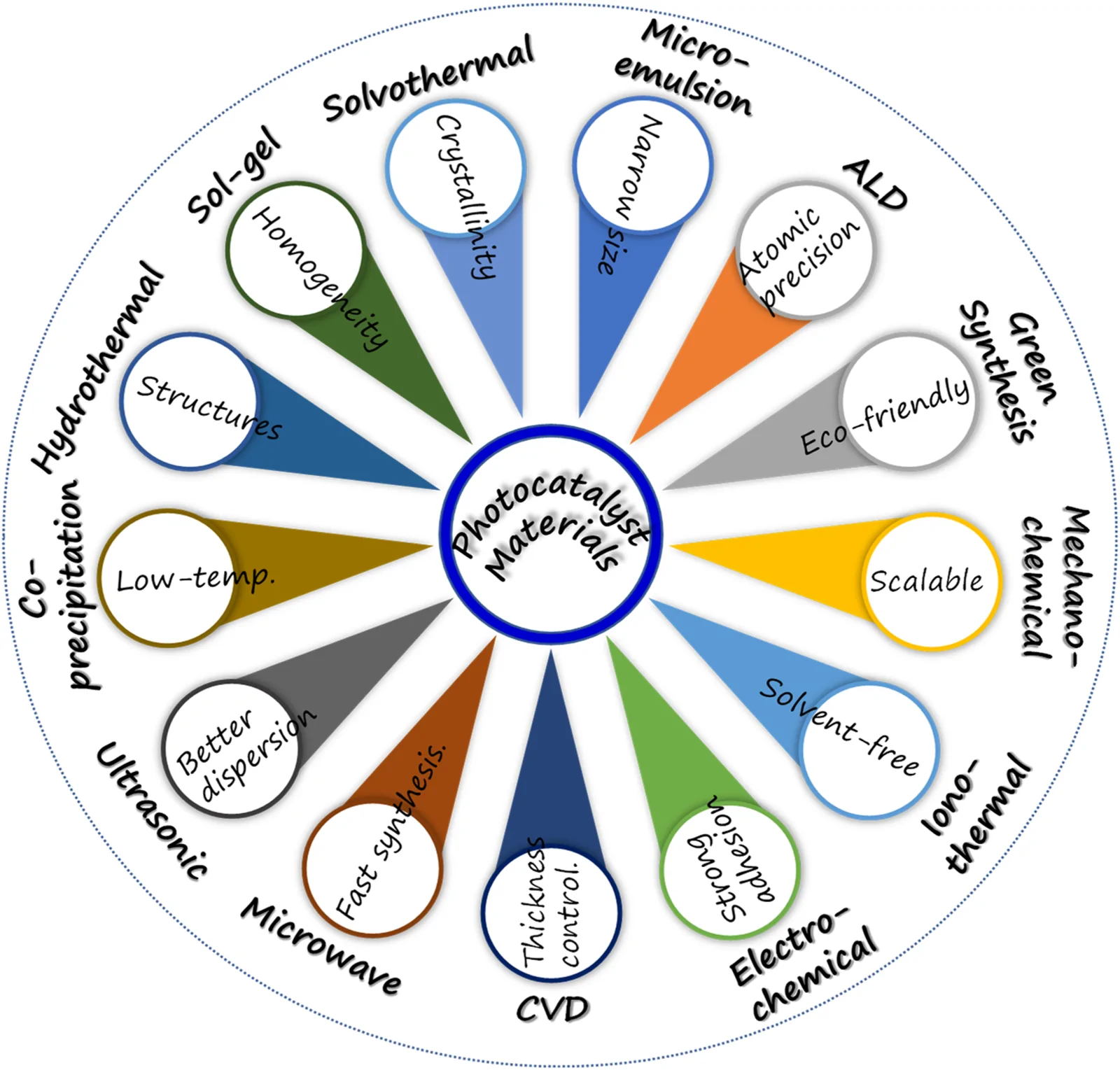
Schematic representation of widely used photocatalyst synthesis routes.

**Table 1:** Representative photocatalytic materials synthesized through different methods, highlighting their photoactive regions, photocatalytic applications, and key performance advantages reported in studies

S. No.	Photocatalyst	Synthesis method	Photoactive region	Application	Performances/Advantages	Ref.
1	TiO_2_ nanotube arrays	Anodization of Ti foil in fluoride-containing electrolyte	UV to near-visible (up to ∼400 nm)	MB degradation	Free-standing TiO_2_ NTs show superior MB degradation *vs.* nanoporous films due to larger contact area and fewer defects	49
2	ZnO nanoflower	Hydrothermal	UV	RhB degradation	Shows one of the highest reported degradation rate constants for bare ZnO; morphology and oxygen vacancies enhance activity, and excellent recyclability	50
3	g-C_3_N_4_ (bulk)	Microwave heating	Visible	MB, MO, & RhB degradation	The layered π-conjugated structure improves the charge transfer and provides active surface sites for photocatalytic reactions	51
4	MoS_2_/CdS hybrid	Hydrothermal MoS_2_ deposition on CdS	Visible	H_2_ evolution from water	H_2_ evolution rate is 12 times higher than that of pure CdS; improved stability over 16 h	52
5	Bi_2_S_3_/MOF-808 composite	Microwave-assisted synthesis of MOF-808, and then *in situ* growth of Bi_2_S_3_	Visible	CO_2_ reduction/dye degradation	Enhanced visible-light reduction *vs.* pure Bi_2_S_3_ or MOF-808 owing to high MOF surface area and better adsorption	53
6	Co-, Fe- and Ni-MOF-74	Hydrothermal	Visible	MB degradation	Co-MOF-74 outperforms Fe/Ni analogues; valence fluctuations of Co more effectively activate peroxymonosulfate	54
7	ZnO–TiO_2_ nanocomposite	Sol–gel/coprecipitation	UV-visible	Organic pollutant degradation	Combined PDS activation; ZnO generates more SO_4_--than TiO_2_, enhancing degradation kinetics	55
8	ZnO microrod-rGO	Hydrothermal	Visible	Phenol degradation	ZnO-rGO degrades ∼84.2% phenol *vs.* lower activity for bare urchin-like ZnO; graphene improves charge separation and surface area	56
9	Ag_2_O–ZnO (ZAO25)	Impregnation of ZnO with AgNO_3_ followed by calcination	Visible	Methylene blue (MB) degradation	∼96.2% MB removal in 30 min; rate constant ∼0.248 min^−1^, ∼26.7 times higher than that of pristine ZnO.	57
10	CuInS_2_/g-C_3_N_4_ (Z-scheme)	Hydrothermal + exfoliation	Visible	Tetracycline hydrochloride	2D/2D Z-scheme structure enhances charge separation and degradation efficiency; high figure of merit	58
11	γ-Fe_2_O_3_/g-C_3_N_4_	Wet-impregnation + calcination	Visible	Tetracycline hydrochloride	Enhances the transfer and separation of photogenerated charge carriers	59
12	Co_3_O_4_/g-C_3_N_4_ Z-scheme	Hydrothermal	Visible	Tetracycline hydrochloride	Improved the transfer and separation of photogenerated charge carriers	60
13	ZnO–CdS	Chemical bath deposition	Visible	H_2_ evolution/dye degradation	Coupling CdS with ZnO improves visible-light absorption and charge separation; enhanced photocurrent and H_2_ yield	61
14	ZnO-graphene	Solution mixing/reduction	UV-visible	Dye degradation	Graphene enhances electron transport and surface area; higher MB/RhB degradation *vs.* bare ZnO.	62
15	ZnO–SnO_2_	Sol–gel	UV-visible	CO_2_ reduction	SnO_2_ improves electron withdrawal from ZnO, enhancing CO_2_ photoreduction performance	63
16	ZnO-WO_3_	Hydrothermal + annealing	UV-visible	Organic pollutant degradation	WO_3_ narrows the ZnO bandgap and improves visible-light response; synergistic degradation of organics	64
17	ZnO–Fe_2_O_3_	Co-precipitation	Visible	Methylene orange/dye degradation	Z-type heterojunction enhances visible-light activity and cycling stability	65
18	ZnO–MnO_2_	Redox-precipitation	UV-visible	Antibiotic degradation	MnO_2_ improves charge-separation and surface–redox reactions; high activity for sulfonamides	66
19	ZnO–CeO_2_	Hydrothermal	UV-visible	Phenol degradation	CeO_2_ provides oxygen storage and defect sites; enhanced radical generation and phenol removal	67
20	ZnO–La_2_O_3_	Coprecipitation	UV-visible	Organic pollutant degradation	Lanthanum sites improve adsorption and surface reactions; stable over multiple cycles	68
21	ZnO–Bi_2_O_3_	Hydrothermal	Visible	Dye degradation	Visible-light-active heterojunction; high MB degradation under solar-simulated light	69
22	ZnO–In_2_O_3_	Sol–gel	UV-visible	CO_2_ reduction	In_2_O_3_ improves conductivity and CO_2_ adsorption; enhanced CO yield under UV-visible irradiation	70

### 4.1 Sol – gel method

The sol–gel method is a widely utilized and versatile wet chemical technique for the synthesis of a broad spectrum of photocatalytic materials. The parameters including pH, solvent viscosity, water-to-alkoxide ratio, and calcination temperature allow precise control over the resultant material properties such as crystallinity, porosity, particle size, and surface area, which strongly influence photocatalytic performance.^71,72^ Due to advantages such as low-temperature processing, cost-effectiveness, excellent compositional homogeneity, ease of doping, and scalability, the sol–gel technique has been extensively employed in the fabrication of metal oxides, doped semiconductors, thin films, and heterostructured photocatalysts for environmental remediation and solar energy conversion applications.^73^ Furthermore, the high purity and tunable microstructure achievable through sol–gel processing enable enhanced light absorption and efficient charge carrier transport, thereby improving photocatalytic efficiency.^74^

Numerous studies have confirmed the applicability of the sol–gel method in synthesizing active photocatalysts with excellent performance. Chen *et al.* prepared ZnO nanostructures synthesized *via* sol–gel routes using zinc acetate as a precursor to effectively degrade various azo dyes such as methyl orange, Congo red, and Direct Black 38 under UV irradiation.^75^ Moreover, Phin *et al.* synthesized hybrid ZnO/carbon nanotube composites through a modified sol–gel route without organic additives which showed enhanced degradation of methylene blue, emphasizing the method's adaptability for synthesizing nanocomposites tailored for dye removal in aqueous environments.^76^ The Fe_2_O_3_/TiO_2_ mesoporous binary systems fabricated *via* a soft-template sol–gel approach used by Palanisamy *et al.* exhibited superior degradation of 4-chlorophenol under solar light due to extended absorption in the visible region, enabled by iron oxide doping.^77^

The sol–gel synthesized photocatalysts have been used in the degradation of pharmaceutical pollutants such as amoxicillin and diclofenac and in the hydrogen evolution reaction.^78^ A notable example includes the fabrication of WO_3_ modified TiO_2_ photocatalysts, where the introduction of WO_3_ into the TiO_2_ matrix *via* sol–gel processing not only improved visible-light absorption by lowering the bandgap from 3.2 eV to approximately 2.6 eV but also facilitated electron–hole separation, enhancing photocatalytic efficiency. In this study, the removal efficiency of amoxicillin reached 64% after 700 °C calcination with a catalyst loading of 0.1 g L^−1^.^79^ Likewise, nitrogen and tungsten co-doped TiO_2_ synthesized through the sol–gel method showed improved particle dispersion and photocatalytic activity, which were confirmed by TEM imaging and XRD analysis, indicating finer crystallites and homogeneous surface morphology.^80^

Despite its many advantages, this method has a few limitations. It requires precise control over synthesis parameters, and deviations may lead to non-uniform particle size distribution or incomplete gelation. Furthermore, post-synthesis calcination can sometimes lead to particle agglomeration or loss of surface area, particularly if sintering temperatures are too high. It was reported that the modification of TiO_2_ with WO_3_ led to bandgap narrowing and excessive sintering reduced surface area and photocatalytic activity due to grain growth.^79^ Materials such as ZnO, TiO_2_, Fe_2_O_3_, and WO_3_, and hybrid systems with carbon nanotubes or reduced graphene oxide have all been successfully synthesized with this method and shown to be effective in degrading diverse organic pollutants and enhancing visible-light-driven photocatalytic processes.^81^

### 4.2 Solvothermal method

The solvothermal method is another powerful and widely used technique for the synthesis of nanostructured photocatalysts with excellent control over particle morphology, size, crystallinity, and surface area, which are key factors directly influencing photocatalytic performance.^82,83^

In recent studies, titanium dioxide nanoparticles synthesized *via* the solvothermal method using titanium tetraisopropoxide and ethanol resulted in anatase-phase structures with spherical morphologies and narrow size distributions, offering improved photocatalytic activity for organic pollutant degradation under UV and visible light.^84^ This method has proven particularly effective for bandgap engineering and improving light-harvesting capability. It is reported that TiO_2_ nanosheets synthesized *via* the solvothermal approach using DMF as a solvent exhibited a significant redshift in the absorption edge, with a bandgap reduced from 3.22 eV (spherical TiO_2_ nanoparticles) to a range of 2.62–2.85 eV for the nanosheets.^85^ This transformation enhanced their visible-light-driven photocatalytic degradation of dyes such as rhodamine B, with studies demonstrating up to 90% degradation under UV light within one hour when employing ZnO microspheres fabricated by similar solvothermal protocols using sodium dodecyl sulfate (SDS) as a surfactant. This method has also been used for hybrid and heterostructure materials. The fabrication of WO_3_/BiVO_4_/BiOCl ternary heterojunctions through a one-pot solvothermal synthesis significantly improved charge separation and migration efficiency, leading to approximately 2-fold and 1.5-fold higher RhB photodegradation under visible light when compared to BiVO_4_ and BiVO_4_/BiOCl binary systems, respectively.^86^ Also, the La-WO_3_/SrTiO_3_ heterojunctions synthesized by this method achieved complete degradation of methyl orange under visible light within 75 minutes and maintained over 90% efficiency after five cycles, demonstrating high stability and reusability.^87^

In addition to oxide-based photocatalysts, the solvothermal method has been used for fabricating composite materials incorporating carbon-based components. Cu_2_O-reduced graphene oxide composites prepared by this method exhibit enhanced surface area and light absorption, with RhB degradation efficiencies exceeding 85% under visible light after multiple cycles.^88^ The presence of rGO not only suppressed charge carrier recombination but also facilitated electron transport, thereby improving photocatalytic kinetics. The graphene/CdS nanocomposites synthesized by this method demonstrated superior hydrogen evolution capabilities due to the uniform dispersion of CdS nanoparticles over graphene sheets.^89^

The solvothermal method has numerous merits like high product purity, excellent crystallinity, and the potential for scalable production but it also has a few challenges. Notably, the sealed nature of autoclave systems prevents real-time monitoring of reaction conditions such as pressure and temperature, which can affect reproducibility. This method requires expensive equipment and poses potential safety concerns due to the high-pressure environment.^41^ Environmental considerations are also pertinent, especially when using toxic solvents like DMF, prompting research toward more benign alternatives. Materials including TiO_2_, ZnO, WO_3_, BiVO_4_, BiOCl, SrTiO_3_, Cu_2_O, CdS, and various MOFs have all been synthesized successfully by this method.^90^ Their applications span from photodegradation of dyes and pharmaceuticals to hydrogen evolution and CO_2_ reduction, showing this method's relevance in both environmental and energy domains.^91^

### 4.3 Hydrothermal method

The hydrothermal method is another technique employed for the synthesis of nanostructured photocatalysts.^92^ The method's flexibility allows fine-tuning of critical synthesis parameters such as temperature, pressure, reaction time, precursor concentration, and pH, which significantly influence the crystallinity, particle morphology, and surface chemistry of the synthesized materials. These attributes are vital for optimizing light absorption and charge separation which are key factors in efficient photocatalysis. This synthesis has been widely used for preparing a range of oxide-based photocatalysts, notably WO_3_, TiO_2_, ZnO, and various metal–organic frameworks (MOFs). WO_3_ nanostructures synthesized by hydrothermal methods exhibit diverse morphologies ranging from monoclinic to orthorhombic phases with bandgaps typically between 2.5 and 3.3 eV depending on the phase and crystallinity.^93^ Enhanced control over phase formation and particle size has allowed researchers to design micro-flower-shaped WO_3_, hexagonal rods, and orthorhombic nanosheets, optimizing their performance in visible-light-driven photocatalysis. The fabrication of graphene-bridged WO_3_/MoS_2_-rGO composites using hydrothermal processing achieved over 90% degradation of rhodamine B under visible light within 120 minutes.^94^ SEM and TEM imaging confirmed well-dispersed flower-like WO_3_ interacting with interwoven MoS_2_ nanosheets supported on rGO, enabling efficient charge transfer and visible light absorption.^93^

This method has shown exceptional efficacy in the preparation of MOFs and their composites like NH_2_-Uio-66(Zr) nanoparticles synthesized *via* hydrothermal treatment at 120 °C for 24 hours which were subsequently hybridized with ZnO to form ZnO/NH_2_-Uio-66 composites. These exhibited outstanding performance in tetracycline degradation due to the large surface area and porous architecture of the MOF scaffold.^95^ Also, MIL-101(Cr)–Ag composites synthesized by combining hydrothermally prepared MIL-101(Cr) with silver nanoparticles displayed efficient size-controlled morphology ranging from 80 to 800 nm, demonstrating enhanced activity in the removal of organic pollutants.^17^ The hydrothermal technique stands out due to its simplicity, cost-effectiveness, scalability, and compatibility with green chemistry principles. It allows for one-step synthesis under relatively mild conditions without requiring organic solvents or high energy input, reducing environmental and economic costs. These photocatalysts generally possess high particle purity, excellent dispersion, and tunable porosity, resulting in enhanced photocatalytic efficiency. A comparative study on TiO_2_–ZnO binary systems synthesized hydrothermally revealed significantly improved structural and surface properties, which translated into higher photocatalytic and antibacterial activity against waterborne pathogens and dye contaminants.^96^

This method has challenges such as relatively long reaction durations and batch limitations, and difficulties in monitoring reaction progress within sealed autoclaves can restrict scalability for industrial applications.^97^ Moreover, achieving uniform doping or precise morphology may still require post-treatment or the integration of complementary synthesis techniques. But still hydrothermal synthesis remains a key tool for tailoring photocatalysts, enabling the creation of nanostructures with morphologies such as nanorods, spheres, sheets, and hollow architectures with controlled bandgap energies suited for visible-light-driven reactions.^98^ Generally, materials synthesized *via* this approach including WO_3_, ZnO, TiO_2_, and diverse MOF-based hybrids have demonstrated robust efficiency in water purification, dye degradation, antibacterial treatment, hydrogen evolution, *etc*.^91^

### 4.4 Co-precipitation method

The co-precipitation method is a widely adopted approach for synthesizing a variety of photocatalysts, particularly mixed metal oxides and layered double hydroxides (LDHs), due to its simplicity, cost-effectiveness, and excellent compositional control.^99,100^ This technique enables the formation of homogeneously distributed multicomponent systems under ambient or moderately elevated temperature conditions, offering precise control over stoichiometry, crystal size, morphology, and particle dispersion.^99^ The physicochemical properties of the resulting photocatalysts, including crystallinity, porosity, surface area, and defect concentration, are strongly influenced by synthesis parameters such as pH, ionic strength, precipitation rate, aging time, and post-synthesis calcination treatment,^100,101^ making this method highly adaptable for photocatalytic applications.

In recent studies, WO_3_/Fe_3_O_4_ nanocomposites synthesized *via* co-precipitation have shown promising activity toward the photodegradation of thiacloprid, a neonicotinoid insecticide.^102^ Four different WO_3_/Fe_3_O_4_ samples were prepared by varying the WO_3_ content, and the optimized catalyst exhibited a degradation efficiency 2.2 times higher than that of pristine Fe_3_O_4_ under both UV and simulated solar light. The enhanced performance was attributed to the synergistic interaction between magnetite and hematite phases of Fe_3_O_4_, which facilitated efficient charge separation and light absorption. Nitrogen physisorption studies showed that the incorporation of WO_3_ contributed to a mesoporous structure with increased specific surface area, critical for the photocatalytic process. This method involves the fabrication of TiO_2_ nanoparticles doped with cobalt. In this case, the doping was achieved under alkaline conditions using ammonia, followed by thermal annealing at 500 °C. The resulting 6% Co-doped TiO_2_ nanocrystals demonstrated a visible-light photodegradation efficiency of nearly 80% for methyl orange, outperforming undoped TiO_2_. The improved activity was linked to reduced particle size, better dispersion, and enhanced light absorption in the visible region.^103^ Likewise, Mahendran *et al.* studied Fe-doped TiO_2_ nanoparticles synthesized *via* co-precipitation which showed significant activity for the degradation of Acid Scarlet 3R dye, indicating that co-precipitation is particularly suitable for incorporating transition metals into semiconductor matrices for bandgap tuning.^104^

This method has also been successfully applied to the synthesis of metal–organic frameworks and their composites. One-step solution precipitation strategies have yielded various MOF morphologies, including rhombic dodecahedral and pitaya-like structures of ZIF-67, depending on the solvent used during synthesis.^105^ These MOFs, when used as precursors for nitrogen-doped nanoporous carbon materials, showed excellent adsorption and photocatalytic performance for degradation of antibiotics such as tetracycline.^106,107^ Moreover, ZnO/SnO_2_ honeycomb nanocomposites modified with TiO_2_*via* co-precipitation exhibited outstanding performance in degrading endocrine-disrupting chemicals like bisphenol A and 2,4-dichlorophenol, demonstrating the versatility of this method in generating complex heterostructures.^108^

This method provides an efficient, lower cost pathway for synthesizing a diverse array of photocatalysts including metal oxides, doped semiconductors, and MOF-based hybrids. Its ability to control particle composition and morphology under relatively mild conditions makes it more attractive for large-scale photocatalyst production. But this method has a few limitations. The formation of amorphous or poorly crystalline phases can occur if post-synthesis thermal treatment is insufficient, and the homogeneity of doping may suffer without rigorous pH control. Also, excessive calcination to improve crystallinity may lead to particle agglomeration and phase segregation, which can compromise photocatalytic efficiency. It is reported that in the synthesis of ZnO/SnO_2_/TiO_2_ composites, increasing the calcination temperature was found to reduce photocatalytic activity due to the formation of inactive phases such as Zn_2_SnO_4_ and ZnTiO_3_.^108^ The photocatalysts produced using this method have shown excellent performance in environmental remediation, particularly in the photodegradation of dyes, pharmaceuticals, and persistent organic pollutants under visible and UV irradiation.^109,110^

### 4.5 Chemical vapour deposition method

The chemical vapour deposition method is an advanced and versatile technique widely employed for synthesizing photocatalytic materials in the form of thin films and coatings with controlled morphology, composition, crystallinity, and thickness.^111,112^ Unlike conventional wet-chemical approaches, CVD involves chemical reactions of volatile gaseous precursors on heated substrates, leading to the deposition of highly uniform and adherent solid films. The high purity of the deposited layers, excellent thickness control, scalability, and conformal coating capability on complex geometries make CVD particularly attractive for fabricating photocatalysts with large surface-to-volume ratios and enhanced charge transport properties.^112^ In this method, parameters such as precursor type, substrate temperature, carrier gas flow, and pressure can be fine-tuned to influence the nucleation and growth of nanostructured films.^111–113^

Its derivatives, such as plasma-enhanced CVD and aerosol-assisted CVD,^114,115^ further extend its applicability to temperature-sensitive substrates and large-area film deposition with improved uniformity. This method has been proved to be quite effective in fabricating titanium dioxide with controlled nanoscale architecture for photocatalytic applications. Taylor *et al.* synthesized TiO_2_ films using aerosol-assisted CVD with titanium isopropoxide as the precursor and acetylacetone as a stabilizing agent and the films exhibited well-defined nanocrystalline morphology with particle sizes in the range of 10–25 nm. These coatings were efficient in degrading Resazurin dye under UV irradiation, with the aerosol modification aiding in reducing reaction temperatures and improving particle dispersion.^116^ In another study, TiO_2_-CNT-Ag ternary composite films were deposited using plasma-enhanced CVD. This approach provided strong interfacial adhesion between components, leading to significantly enhanced photocatalytic degradation of organic pollutants. The composite exhibited higher visible light absorption, primarily due to localized surface plasmon resonance of Ag and the broad-spectrum response of the CNT matrix.^117^

This method is particularly advantageous for producing oxide coatings such as ZnO, WO_3_, and TiO_2_ on glass, silicon, or ceramic substrates. In applications targeting photocatalytic NO_*x*_ remediation or water purification, the deposition of nanostructured TiO_2_ films on transparent substrates has enabled the development of self-cleaning and air-purifying surfaces. Such films demonstrate consistent photodegradation of airborne contaminants like nitrogen oxides and volatile organic compounds.^118^ However, despite its precision and scalability, this method also faces certain challenges. The higher processing temperature required for precursor decomposition limits its application to thermally stable substrates. Also, the handling of volatile and sometimes toxic precursors requires rigorous safety measures and specialized equipment. To address these limitations, modifications such as lower temperature plasma enhancement and precursor dilution techniques have been developed.^119^ These allow broader substrate compatibility and mitigate thermal damage during film growth while preserving film quality and photocatalytic function. This technique plays a vital role in the fabrication of advanced photocatalysts with desirable characteristics like higher surface uniformity, strong adhesion, tuneable thickness, and excellent stability.^120^ It is especially suited for creating nanostructured films of TiO_2_, ZnO, and other semiconductor oxides with applications in pollutant degradation, antibacterial surfaces, self-cleaning windows, and photoelectrochemical hydrogen production.^121^

### 4.6 Microwave method

The microwave-assisted method is an efficient and rapidly emerging technique for fabricating high-performance photocatalytic materials due to its advantages of rapid heating, energy efficiency, shorter reaction times, and uniform volumetric heat distribution.^122^ In this method, microwave irradiation directly interacts with the reaction medium, enabling instantaneous and homogeneous heating throughout the material, which facilitates controlled nucleation and crystal growth.^100^ As a result, microwave-assisted synthesis often produces nanomaterials with uniform morphology, narrow particle-size distribution, enhanced crystallinity, higher surface area, and improved photocatalytic activity compared with conventional heating methods.^100^ Furthermore, microwave heating generally operates at lower temperatures and significantly reduces synthesis duration without requiring high-pressure autoclaves, making it a safer, environmentally friendly, and sustainable approach for photocatalyst fabrication.^40^ Owing to these advantages, microwave-assisted synthesis has been extensively utilized for preparing metal oxides, doped semiconductors, quantum dots, and heterostructured photocatalysts for applications in pollutant degradation, hydrogen evolution, and solar energy conversion.^112^

Several studies have demonstrated the effectiveness of this method for the synthesis of metal–organic frameworks (MOFs) and semiconductor composites. In a notable study by Xu *et al.*, Fe-doped Uio-66(Zr) photocatalysts were synthesized using a two-step method, where the Fe^3+^ ions were successfully incorporated into the MOF structure under microwave irradiation at 85 °C for just 30 minutes. The resulting polyhedral Fe-Uio-66 photocatalyst exhibited a homogeneous morphology and efficient light absorption, indicating the effective integration of Fe ions into the Uio-66 framework.^123^ Similarly, Crake *et al.* employed the microwave method to synthesize TiO_2_/NH_2_-Uio-66 composites, where the reaction mixture was irradiated at 80 °C for one hour. The small NH_2_-Uio-66 nanoparticles uniformly coated on the TiO_2_ surface facilitated enhanced interfacial charge separation, thus improving photocatalytic activity under visible light.^124^

This method has been effectively applied to semiconductor–graphene composites besides MOF-based systems. TiO_2_/graphene and ZnO/graphene oxide (GO) nanostructures synthesized under various microwave irradiation times showed significant improvements in photocatalytic performance due to enhanced charge transport and light absorption properties. It is reported that GO-ZnO heterostructures synthesized at different irradiation durations (3.0, 4.5, and 7.5 minutes) led to the formation of microcubes, nanoflakes, and nanoneedles, respectively.^125^ These distinct morphologies tailored based on irradiation time enabled control over surface area and active sites, which directly influenced dye degradation efficiency.

Furthermore, NH_2_-MIL-125(Ti) and its Cu-modified composite were synthesized within three minutes at 150 °C using a microwave system, demonstrating the remarkable speed and efficiency of this method. The rapid crystallization process led to disk-like nanostructures with high photocatalytic potential for pollutant degradation.^126^ The Cu-doped composite, in particular, benefited from the synergistic effect of Ti-based MOFs and transition metal active sites, promoting enhanced light absorption and charge separation. Furthermore, 2D lanthanide-based MOF composites fabricated under microwave irradiation at 231 W showed thickness-dependent photocatalytic performance, highlighting the method's suitability for engineering morphology-sensitive materials.^127^

Despite its many advantages, the microwave method has limitations, primarily related to the difficulty in controlling particle size and uniformity during rapid heating. Variations in dielectric properties of the reactants and solvents can lead to hot spots or uneven nucleation, which may impact the reproducibility of the synthesized materials.^128^ Also, scaling up the microwave-assisted processes remains a technical hurdle due to limitations in uniform energy distribution across larger volumes. It has been successfully employed for the synthesis of various advanced materials including MOFs, TiO_2_, ZnO, CuInGaSe_2_, and ZnS-based nanostructures, often in combination with carbon-based materials like graphene oxide and reduced graphene oxide. These composites have demonstrated enhanced photocatalytic activity in degrading pollutants such as methylene blue, rhodamine B, and reactive black dyes, and also in the hydrogen evolution reaction under visible or solar light irradiation.^129,130^

### 4.7 Ultrasonic method

The ultrasonic method, also known as sonochemical synthesis, is a highly effective and energy-efficient strategy for fabricating advanced photocatalysts, especially those involving composite or hybrid nanomaterials. The technique leverages the phenomenon of acoustic cavitation, the formation, growth, and implosive collapse of microscopic bubbles in a liquid medium under ultrasonic irradiation.^131^ These cavitation events generate localized hot spots with extreme temperatures and pressures leading to the formation of highly reactive species and promoting rapid chemical reactions.^132^ Such intense energy enables the synthesis of photocatalytic materials with improved crystallinity, high surface area, uniform dispersion, and enhanced interfacial contact, all of which are critical parameters for superior photocatalytic performance.^133^

This synthesis has been successfully applied to the fabrication of various photocatalyst composites, including metal oxides, graphene-based hybrids, and metal–organic frameworks (MOFs). ZnO/reduced graphene oxide (rGO) composites were synthesized *via* a simple ultrasonic mixing process, where the uniform distribution of ZnO nanoparticles on the rGO sheets was achieved due to cavitation-assisted dispersion.^134^ The resulting composite exhibited a narrowed bandgap and improved charge transport which enhanced visible-light absorption and photocatalytic efficiency. Lal *et al.* synthesized neodymium-doped TiO_2_ using a combined sol–gel and ultrasonic method and the resultant nanostructure demonstrated photocatalytic degradation efficiencies of 99.11% and 96.42% under UV and solar light, respectively, attributed to the smaller particle size and increased surface area enabled by ultrasonic treatment.^135^

This method has shown exceptional promise in engineering high-performance MOF photocatalysts. Abazari *et al.* prepared g-C_3_N_4_/Ti-MIL125-NH_2_ composites through a 20 minute ultrasonic treatment in a methanol-DMF mixture. This hybrid material featured enhanced light absorption and a larger surface area, leading to efficient degradation of 4-nitrophenol.^136^ Hlophe and his colleagues used sonication-assisted synthesis of MIL-125(Ti)/black phosphorus (BP) composites which yielded uniformly distributed MOF nanocrystals on BP sheets, improving both structural stability and conductivity.^137^

Moreover, this method has enabled the synthesis of unique morphologies such as when Zhao and co-workers fabricated the polyhedral ZIF-8-derived nitrogen-doped graphene (ZNG) composites. The incorporation of sonication not only facilitated homogeneous nucleation but also assisted in post-synthesis modifications like carbonization, further boosting photocatalytic efficiency.^138^ In a related study, Sharotri *et al.* synthesized Mn–N co-doped TiO_2_ using a cavitation-induced approach that exploited the intense localized energy of ultrasonic irradiation to create oxygen vacancies. The resulting photocatalyst demonstrated enhanced light absorption and reactive oxygen species (ROS) generation, ultimately accelerating the photodegradation of organic pollutants.^139^

The ultrasonic method offers a green, rapid, and effective route for synthesizing nanostructured photocatalysts with superior physicochemical properties such as high surface area, improved crystallinity, reduced particle size, and superior dispersion stability.^133^ The method demonstrated efficacy in pollutant degradation, antibacterial activity, and visible-light-driven photocatalysis, which play a critical role in the scalable development of next-generation photocatalytic materials.^140^ Also, the ultrasonic method has certain limitations. The uniformity and reproducibility of particle size and morphology can be challenging to control due to the stochastic nature of cavitation.^133^ Additionally, prolonged sonication can lead to re-agglomeration or surface degradation in some sensitive systems.^140^ Energy consumption, although generally lower than in thermal methods, must also be optimized to prevent inefficient cavitation. However, these limitations can often be mitigated by optimizing parameters such as sonication power, frequency, solvent system, and reaction time.

### 4.8 Electrochemical method^122,131,141^

The electrochemical method particularly electrochemical deposition (ECD) is a promising technique in the synthesis of photocatalytic materials due to its simplicity, higher precision, and ability to control material composition and morphology.^141,142^ This method involves the reduction of metal ions from an electrolyte solution onto a conductive substrate under an applied electrical potential. The electrochemical environment facilitates the formation of thin films, nanoparticles, and nanostructured composites with tailored properties, which are critical for enhancing photocatalytic performance.^141^ Moreover, the room-temperature and aqueous processing conditions often employed in ECD make it an environmentally benign and cost-effective alternative to high-temperature or solvent-intensive methods.^143^ This method has the ability to construct layered or hierarchical photocatalytic architectures with higher purity and excellent adhesion to substrates.

Cirak *et al.* synthesized ZnO-decorated TiO_2_ nanotube arrays using a tandem process combining anodic oxidation and chemical bath deposition.^144^ The resulting TiO_2_/ZnO hybrid exhibited significantly enhanced photocatalytic degradation of rhodamine B compared to pure TiO_2_ or ZnO, primarily due to improved charge carrier separation and increased surface area. In one study, Zn^2+^ ions were electrochemically deposited onto reduced graphene oxide sheets, yielding a uniformly dispersed ZnO network that facilitated efficient charge transfer and suppressed electron–hole recombination. The synthesized composite exhibited a notable improvement in degradation efficiency for dyes such as methylene blue and methyl orange under UV and visible light due to the uniform nucleation and the intimate contact between ZnO and the graphene matrix, which promoted synergistic photocatalytic effects.^145^ Beyond oxide-based systems, electrochemical techniques have enabled the integration of metal nanoparticles into semiconductor frameworks. Noble metals like Ag and Au have been electrodeposited onto TiO_2_ and ZnO substrates to create plasmonic photocatalysts that absorb more visible light and enhance the rate of interfacial charge transfer.^146^ Such approaches have been shown to significantly increase hydrogen production rates in photoelectrochemical water splitting and enhance photocatalytic degradation rates for recalcitrant pollutants.^147^

The electrochemical method suffers from certain limitations. The deposition rate and morphology are highly sensitive to parameters such as current density, electrolyte composition, and substrate geometry, requiring meticulous optimization.^148^ Also, ECD is generally limited to conductive substrates, which can restrict material choice or necessitate complex substrate modification. Achievement of uniform large-area coatings or multilayer heterostructures may demand intricate electrode design or the use of supplementary deposition steps which poses a big challenge.^147^ This method offers a precise, scalable, and environmentally friendly route for the synthesis of advanced photocatalysts. It enables the fabrication of diverse photocatalytic systems including ZnO, TiO_2_, metal–semiconductor hybrids, and graphene-based composites which have shown high efficiency in degrading dyes, pharmaceuticals, and persistent organic pollutants, as well as in photocatalytic hydrogen evolution.^145^

### 4.9 Mechanochemical method

The mechanochemical method, particularly *via* ball milling, has evolved as a sustainable, solvent-free, and highly productive route for synthesizing photocatalytic materials.^149^ This technique relies on the application of mechanical energy through grinding or milling processes to induce chemical reactions between solid-state precursors.^150^ It has several advantages like simplicity, lower energy input, room-temperature operation, and the ability to produce nanomaterials with higher surface area and tunable surface defects to enhance photocatalytic activity.^112^ This technique avoids toxic solvents and complex processing, aligning well with green chemistry principles. The mechanical force exerted during milling leads to particle size reduction, increased crystallinity, surface activation, and sometimes phase transformation, all of which are beneficial for optimizing the photocatalytic properties of the materials.

Wu *et al.* reported mechanochemical synthesis which includes the preparation of TiO_2_ nanoparticles doped with Gd^3+^ and modified with Gd_2_O_3_.^151^ This ball milling of systems introduced oxygen vacancies and lattice distortions, which serve as active sites for photocatalysis and trap photogenerated charge carriers, thereby reducing recombination rates and enhancing efficiency. The resulting nanocomposites exhibited superior activity in the degradation of organic pollutants compared to undoped TiO_2_. The ZnO/biochar nanocomposites fabricated by Yu *et al.* through mechanochemical means demonstrated excellent performance in methylene blue degradation.^152^ The enhanced efficiency was attributed to the increased meso- and macroporosity of the nanocomposite, induced by mechanical milling, which facilitated higher pollutant adsorption and improved light-driven reactivity. This method has been used in the synthesis of binary and ternary oxide systems. In one study, TiO_2_ was doped with various metal oxides such as Fe_2_O_3_ and CeO_2_*via* high-energy milling, resulting in nanostructures with reduced band gaps and enhanced absorption in the visible region. These photocatalysts showed substantial degradation efficiencies for persistent dyes and antibiotics under both UV and visible light.^153^ The mechanochemical method's capability to create defect-rich surfaces and to induce amorphous-to-crystalline transitions without higher thermal treatment proves particularly beneficial for photocatalytic systems where charge mobility and surface reactivity are key factors.

However, the mechanochemical method also presents certain challenges despite its numerous advantages. Precise control over particle size distribution, morphology, and compositional homogeneity can be difficult because of the stochastic nature of mechanical collisions occurring during the milling process.^150^ Moreover, prolonged milling may induce undesirable phase transformations, excessive amorphization, structural disorder, or over-grinding, which can adversely affect the crystallinity and photocatalytic performance of the synthesized materials.^154^ Another important limitation is the potential contamination arising from wear of the milling media and container materials, particularly when processing highly reactive or sensitive systems.^154^ Nevertheless, several advanced strategies including cryo-milling, inert-atmosphere milling, process control agents, and surface passivation techniques have been developed to improve reproducibility, minimize contamination, and enhance the structural quality of mechanochemically synthesized photocatalysts.^149^ Despite these limitations, modifications such as cryo-milling, use of inert atmospheres, and surface passivation strategies have been explored to enhance the reproducibility and quality of the synthesized materials.^155^ The mechanochemical method offers a green, efficient, and scalable approach to develop advanced photocatalytic materials and its applicability spans a wide range of systems, including doped oxides, oxide-carbon composites, and even metal-free photocatalysts.^149^ The materials have demonstrated excellent performance in pollutant degradation, antibacterial applications, photocatalytic hydrogen evolution and environmental remediation technologies.^156^

### 4.10 Ionothermal method

The ionothermal method has emerged as an advanced and environmentally benign approach for synthesizing photocatalytic materials using ionic liquids (ILs) or deep eutectic solvents (DESs) as both solvents and structure-directing agents.^157–159^ This solvent-free or lower solvent approach combines the benefits of higher thermal and chemical stability, negligible vapor pressure, and tunable solvation environments. The ionothermal route offers precise control over the morphology and composition of photocatalysts particularly in the synthesis of MOFs, zeolites, and porous oxides while reducing the environmental impact associated with traditional organic solvents.^159,160^ This synthesis allows the facilitation of slow nucleation and controlled crystal growth due to the higher viscosity and unique coordination behaviour of ionic liquids which leads to enhanced crystallinity and uniform particle distribution. Such features are critical for promoting light absorption and charge separation during photocatalytic processes. Also, the thermal and chemical resistance of ionic liquids allows for the formation of novel frameworks that may not be accessible *via* aqueous or organic routes.^160,161^

In comparison to hydrothermal or solvothermal routes, there are few published papers on ionothermal synthesis of photocatalysts. The studies suggest that this method is particularly effective in engineering hybrid systems with enhanced photocatalytic properties. Zn-based and Fe-based MOFs synthesized in IL media have demonstrated improved band alignment and lower recombination rates of photoinduced carriers.^161,162^ Furthermore, composites like TiO_2_/IL-MOF hybrids prepared through ionothermal processing have shown exceptional performance in visible-light-driven degradation of dyes and antibiotics.^162^ These improvements are attributed to the synergistic interaction between the IL-functionalized framework and semiconductor components, facilitating efficient charge mobility and pollutant adsorption. However, the ionothermal method also faces certain challenges. The higher cost and limited recyclability of some ionic liquids may impede scalability. Moreover, the removal of residual ILs from the final product often requires additional washing steps or calcination, which may alter the surface chemistry of the synthesized material.^158,159^ However, ongoing research into green and cost-effective IL analogs, such as DESs, is addressing these limitations and enhancing the method's applicability for industrial-scale production.^160,161^ The ionothermal method offers a green, versatile, and tunable platform for the synthesis of advanced photocatalysts with higher crystallinity, porosity, and chemical stability. Its success in fabricating MOFs, zeolite analogs, and semiconductor composites with tailored photoelectronic properties highlights its potential for future applications in water purification, air remediation, and solar fuel generation.^162^

### 4.11 Microemulsion method

The microemulsion method is a technique for the preparation of photocatalytic nanomaterials, particularly for achieving highly uniform particle sizes, controlled morphologies, and superior surface characteristics.^163^ Microemulsions are thermodynamically stable, isotropic liquid mixtures of water, oil, and surfactants that form nanoscale droplets which provide spatial confinement during the nucleation and growth of particles, thus enabling the synthesis of highly dispersed and monodispersed nanomaterials.^164^ This technique has enabled the fabrication of a variety of semiconductor oxides, sulfides, and hybrid materials like TiO_2_, ZnO, and CdS nanoparticles which have demonstrated significantly enhanced photocatalytic activity.^163^ The nanoscale environment within microemulsion droplets restricts uncontrolled particle growth and aggregation, leading to the formation of photocatalysts with greater light-harvesting ability and more effective charge carrier separation. The resulting nanostructures exhibit excellent performance in degrading organic dyes such as MB and RhB, as well as in applications like hydrogen evolution and CO_2_ reduction under solar or visible light irradiation.^112^

Several studies have highlighted the superior performance of photocatalysts synthesized through the microemulsion route. ZnO quantum dots prepared using water-in-oil microemulsions exhibited ultra-small particle sizes below 10 nm due to surfactant-assisted growth restriction, resulting in quantum confinement effects and enhanced photodegradation efficiency toward phenolic pollutants.^165,166^ Similarly, CdS nanoparticles synthesized *via* reverse micelle systems showed excellent colloidal stability and maintained high photocatalytic activity even after multiple degradation cycles.^166^ In many cases, photocatalysts synthesized by the microemulsion technique outperform those prepared by conventional precipitation or sol–gel methods owing to their improved dispersion, smaller particle size, and stronger interfacial interactions.^164^ Despite these advantages, the microemulsion method also faces several challenges. The selection and optimization of surfactants, co-surfactants, oil phases, and solvent ratios are critical for maintaining thermodynamic stability and controlling droplet size.^164^ Furthermore, post-synthesis purification to remove residual surfactants can be complex and may alter the surface chemistry or photocatalytic activity if not performed carefully.^167^ Large-scale production is also limited by the high cost of surfactants and the difficulty in maintaining uniform micellar environments during scale-up processes. Nevertheless, the outstanding control over nanostructure formation and photocatalytic functionality makes the microemulsion method a highly promising strategy for the fabrication of next-generation photocatalytic materials for environmental remediation, water splitting, air purification, and solar fuel generation.

### 4.12 Atomic layer deposition method

The atomic layer deposition method is a precision-oriented, vapor-phase technique that enables the layer-by-layer fabrication of ultrathin films with atomic-scale control over thickness, uniformity, and composition.^168^ The higher degree of control offered by ALD permits the engineering of heterostructures and defect-rich interfaces that are essential for optimizing charge separation and improving light harvesting in semiconductor-based photocatalysts. Its versatility spans the deposition of binary, ternary, and doped oxides, as well as hybrid systems, making it particularly attractive for tailoring band alignment and surface energetics in photocatalysis. Recent advancements have shown that ALD can significantly enhance the performance of TiO_2_-based photocatalysts. In a study by Cao *et al.*, Fe_2_O_3_ was deposited as a surface modifier on TiO_2_ nanoparticles using ALD, leading to a marked improvement in visible-light photocatalytic activity.^169^ The composite exhibited superior degradation performance toward Congo red dye under visible light, clearly demonstrating the efficacy of ALD in tuning photocatalytic interfaces. Moreover, the deposition of sub-nanometer films in ALD avoids agglomeration issues common with wet-chemistry methods and ensures better dispersion of catalytic sites. Also, TiO_2_ thin films deposited *via* ALD were immobilized on porous substrates to create robust photocatalytic coatings with higher surface area and light transparency.^170–172^ These films exhibited consistent photocatalytic activity over multiple cycles for the degradation of RhB and MB dyes. ALD was also reported to deposit ZnO and WO_3_ films with precisely controlled thicknesses on nanostructured templates, resulting in enhanced optical absorption and longer carrier lifetimes which are two crucial attributes for photocatalytic hydrogen evolution and organic pollutant degradation.^171–173^

The ALD technique requires specialized equipment and a relatively slow growth rate per cycle (typically 0.1–0.2 nm) which makes it less favourable for bulk material synthesis.^170^ Moreover, the selection of precursors must ensure adequate volatility and reactivity while minimizing contamination.^171,172^ Generally, TiCl_4_ and water are commonly used for TiO_2_ deposition but residual chlorine can negatively influence the photocatalytic performance if not properly removed.^173^ Nonetheless, recent developments in plasma-enhanced ALD have addressed these concerns by enabling lower temperature processes and improving film purity.^172–174^ This method offers a higher precision, conformal, and tunable synthesis method for photocatalytic materials, particularly suitable for nanostructured and hierarchical systems.^175^ Its ability to engineer interfacial properties at the atomic level has enabled the fabrication of highly active photocatalysts such as Fe_2_O_3_–TiO_2_, ZnO-coated nanorods, and other heterojunctions tailored for pollutant degradation, antibacterial surfaces, and solar-driven hydrogen evolution.^176^

### 4.13 Green synthesis method

The green synthesis method employs biological resources such as plant extracts, bacteria, fungi, algae, and other biomaterials to fabricate photocatalytic nanomaterials under mild and environmentally benign conditions.^177^ In contrast to conventional chemical and physical routes, which often depend on toxic solvents, elevated temperatures, and costly reagents, green synthesis uses naturally occurring phytochemicals or biomolecules as reducing, capping, and stabilizing agents.^178^ This approach is consistent with the principles of green chemistry because it reduces hazardous waste, lowers energy demand, and supports safer and more sustainable nanomaterial production. The development of green composites from agricultural residues is a paramount route in maintaining a sustainable environment.^179^ The key advantage of green synthesis lies in the multifunctional role of biological extracts during nanoparticle formation. Functional groups such as hydroxyl, carboxyl, amino, and phenolic moieties can reduce metal ions, promote nucleation, and regulate particle growth, thereby influencing size, morphology, crystallinity, and surface chemistry. As a result, green-synthesized nanomaterials often show improved stability, enhanced surface reactivity, and favorable biocompatibility, which are valuable for photocatalytic applications.^180^ Among the available bio-based routes, plant-mediated synthesis has received the most attention because it is simple, scalable, non-pathogenic, and readily adaptable to aqueous media.^181^

Recent studies have demonstrated the successful green synthesis of a wide range of semiconductor photocatalysts, including ZnO, TiO_2_, SnO_2_, and CeO_2_ nanoparticles using extracts derived from leaves, seeds, fruits, and roots.^182^ Green-synthesized ZnO nanostructures, in particular, have been extensively studied for solar-driven degradation of dyes and other organic contaminants. For example, ZnO nanoflowers synthesized using *Chlamydomonas reinhardtii* microalgae achieved 90% degradation efficiency against dyes within two hours under sunlight,^182^ while ZnO nanoparticles prepared with *Artocarpus heterophyllus* (jackfruit) leaf extract showed a high photocatalytic degradation efficiency of over 80% for Rose Bengal dye within 60 minutes.^183^ These materials are frequently reported to exhibit narrowed effective band gaps, stronger light absorption, and higher surface defect density, all of which can improve charge separation and overall photocatalytic efficiency.^184^

Microbial-assisted synthesis is another important branch of green nanotechnology. Bacterial species such as *Bacillus amyloliquefaciens*, *Bacillus subtilis*, and *Bacillus licheniformis* have been employed to synthesize TiO_2_ and ZnO nanoparticles with controlled morphology and high catalytic stability. Khan and Fulekar used *Bacillus amyloliquefaciens* to facilitate the biosynthesis of TiO_2_ nanoparticles in the size range of 15–88 nm which showed efficient degradation of Reactive Red 31 dye under UV irradiation.^185^ Likewise, ZnO nanoflowers produced using *Bacillus licheniformis* achieved 83% decolorization of methylene blue in 60 minutes and maintained excellent recyclability over multiple photocatalytic cycles.^15^ Despite its promise, green synthesis still faces several practical limitations. The composition of biological extracts can vary with species, geographic origin, extraction conditions, and storage, which affects reproducibility and batch-to-batch consistency. In addition, precise control over nanoparticle size, crystallinity, and phase purity remains more challenging than in conventional synthetic routes.^186^ Scaling up production while maintaining consistent physicochemical properties is therefore a major obstacle for industrial implementation. The advances in bioengineering and synthetic biology and the integration of green-synthesized nanomaterials with other functional materials, such as graphene or MOFs, offer promising avenues to further enhance photocatalytic efficiency and stability.^165^ To address these issues, future work should focus on standardizing extract preparation, identifying active biomolecules, and integrating green synthesis with controlled post-treatment or composite formation.

This synthesis method offers an eco-friendly, cost-effective, and scalable alternative for producing high-performance photocatalysts. Its ability to generate ZnO, TiO_2_, and related nanomaterials with favorable surface chemistry and strong photocatalytic response makes it especially attractive for dye degradation, water purification, and solar energy applications.^166^ With improved standardization and mechanistic understanding, green synthesis is likely to remain a central strategy in the development of sustainable photocatalytic materials. To provide a holistic understanding of the various synthesis strategies employed for photocatalyst fabrication, it is essential to compare their fundamental characteristics, advantages, limitations, and practical applicability. Each method offers unique benefits in terms of material morphology, crystallinity, scalability, and environmental compatibility, which directly influence photocatalytic performance.^167^

Table 2 presents a concise comparative analysis of these synthesis methods, offering insights into their suitability for specific photocatalytic applications. Different fabrication methods provide different advantages for photocatalytic materials. The suitability of a synthesis route depends on the material type, required morphostructure, photocatalytic application, and scalability. Conventional methods such as sol–gel, hydrothermal, and co-precipitation are widely used because they are simple, cost-effective, and suitable for large-scale production. These methods also provide good control over particle size and crystallinity. In contrast, advanced methods such as atomic layer deposition, electrospinning, microwave-assisted synthesis, and plasma treatment offer better control over defects, interfaces, and nanostructures, which can improve charge separation and photocatalytic performance. However, these techniques usually require expensive equipment, higher energy input, and more complex processing. Therefore, the choice of fabrication method should consider not only photocatalytic efficiency but also reproducibility, stability, cost, and practical scalability. Overall, the most effective synthesis strategy is the one that provides a controlled morphostructure, stable interfaces, optimized defects, and reliable long-term photocatalytic performance.

**Table 2:** Comparative analysis of photocatalyst synthesis methods

Synthesis method	Key features	Advantages	Limitations	Materials synthesized	Photocatalytic applications	Ref.
Sol–gel	Wet-chemical method based on hydrolysis and condensation	Low-cost, good homogeneity, and precise control over composition and morphology	Time-consuming drying and calcination; risk of agglomeration	TiO_2_, ZnO, WO_3_, and Fe_2_O_3_ composites	Dye degradation, H_2_ generation, and air/water purification	51
Solvothermal	Uses organic solvents at high temperature and pressure	Excellent crystallinity, tunable shape/morphology, and good for heterostructures	Requires high-pressure autoclaves; limited scalability	TiO_2_, BiVO_4_, ZnTiO_3_, and WO_3_-based composites	Visible-light photocatalysis, CO_2_ reduction, and dye degradation	62
Hydrothermal	Water-based, high-temperature/pressure synthesis in sealed vessels	Eco-friendly, high purity, and suitable for nanorod/sheet structures	Long synthesis time, batch limitation, and poor *in situ* monitoring	TiO_2_, ZnO, WO_3_, MOFs, and g-C_3_N_4_ composites	Water disinfection, dye degradation, and photocatalytic H_2_ evolution	70
Co-precipitation	Simultaneous precipitation of multiple ions in solution	Simple, scalable, low-temperature synthesis, and homogeneous doping	Poor crystallinity without post-annealing; possible phase separation	TiO_2_/Fe, ZnO, ZrO_2_, MOFs, and ternary oxides	Organic dye degradation; pharmaceutical removal	77
Chemical vapor deposition	Gas-phase deposition *via* precursor reaction on a heated substrate	High-purity, conformal thin films, and atomic-level thickness control	Requires high temp & vacuum systems and costly precursors	TiO_2_, ZnO, TiO_2_ – CNT -Ag, and WO_3_	Self-cleaning surfaces, NOx removal, and solar fuel cells	90
Microwave	Rapid heating *via* microwave irradiation	Fast synthesis, uniform particle formation, and energy-efficient	Difficulty in scaling, hot spot formation, and limited control over particle growth	ZnO, TiO_2_, g-C_3_N_4_, MOFs, and graphene composites	Dye degradation, antibiotic removal, and H_2_ evolution	100
Ultrasonic	Cavitation-based synthesis using high-frequency ultrasound	Room temperature, rapid mixing, and better dispersion and crystallinity	Particle size variability; potential contamination from probe materials	TiO_2_, ZnO, MOFs, and rGO nanocomposites	Visible-light catalysis, antibacterial surfaces, and dye degradation	109
Electrochemical	Electrodeposition or anodization from ionic precursors	Precise thickness control, low temp, and strong adhesion on substrates	Limited to conductive substrates; sensitive to deposition parameters	TiO_2_/ZnO, Ag/ZnO, and graphene–ZnO composites	Photo-electrochemical water splitting and dye removal	120
Mechanochemical	Solvent-free synthesis *via* mechanical grinding or milling	Green, scalable, energy-efficient, and induces defects and doping	Limited control over uniformity and equipment contamination risk	TiO_2_, ZnO, Fe_2_O_3_, and biochar composites	UV/solar-light degradation; antibiotic removal	127
Ionothermal	Uses ionic liquids as solvents and structure-directing agents	Solvent-free, tunable porosity, and high crystallinity	Cost of ILs; residual IL removal challenges	MOFs, TiO_2_/IL composites, and Fe/Zn MOFs	CO_2_ reduction, dye degradation, and solar fuels	137
Microemulsion	Nanoscale reactors formed from water-oil-surfactant systems	Narrow size distribution; morphology control at room temperature	Complicated formulation; surfactant removal post-synthesis	ZnO, TiO_2_, CdS, and Ag-based quantum dots	Dye degradation, hydrogen evolution, and photocatalytic sensors	141
Atomic layer deposition	Layer-by-layer gas-phase deposition *via* surface-limited reactions	Atomic precision, ultra-thin conformal coatings, and excellent interfacial tuning	Slow deposition rate; expensive instrumentation	TiO_2_, Fe_2_O_3_/TiO_2_, and ZnO films	Heterojunctions for H_2_ generation; visible-light catalysis	146
Green synthesis	Biogenic route using plant extracts, microbes, or algae	Eco-friendly, low-cost, biocompatible, and avoids toxic reagents	Reproducibility issues, purification complexity, and slow reaction rates	TiO_2_, ZnO, CeO_2_, SnO_2_, and MOFs *via* plant/bacterial extracts	Antibacterial coatings, dye degradation, and water treatment	155

## 5 Morphostructure of photocatalytic materials

The morphostructure of photocatalytic materials plays a decisive role in governing light harvesting, charge separation, interfacial transport, and adsorption of reactants on the catalyst surface. Because photocatalysis is inherently a surface-mediated process, parameters such as particle size, surface area, porosity, crystallinity, and exposed crystal facets directly influence catalytic kinetics and overall efficiency.^41^ Accordingly, morphological engineering has emerged as a central strategy for optimizing photocatalysts for environmental remediation and solar fuel generation.^46^ Photocatalytic nanomaterials are commonly classified according to dimensionality, including zero-dimensional quantum dots and nanospheres, one-dimensional nanorods, nanowires, nanotubes, and nanofibers, two-dimensional nanosheets and nanoplates, and three-dimensional hierarchical or porous architectures.^68^ Each geometry offers a distinct balance between active-site density, carrier transport distance, and optical response. In general, 0D structures provide high surface-to-volume ratios and quantum confinement effects, 1D architectures facilitate directional charge migration, 2D materials offer short carrier diffusion lengths and large exposed surfaces, while 3D porous structures enhance light scattering and mass transport through interconnected pore networks.

Among these morphologies, hierarchical and defect-engineered architectures often exhibit superior photocatalytic activity because they integrate multiple beneficial features simultaneously. For instance, 3D porous WO_3_ nanostructures have shown enhanced photocatalytic performance due to improved light absorption, larger surface area, and efficient reactant diffusion.^168^ Similarly, ZnO nanostructures with different morphologies such as nanoflowers, nano-urchins, and nanosponges exhibit significantly different photocatalytic efficiencies depending on crystallinity, defect distribution, pore development, and annealing conditions.^68^ These observations confirm that photocatalytic performance cannot be explained solely by morphology, but rather by the combined influence of structural features, defect chemistry, and surface properties. Therefore, future photocatalyst design is increasingly focused on hybrid, hierarchical, and defect-engineered morphologies capable of simultaneously improving adsorption, charge separation, visible-light utilization, and redox reactivity.

An overview of different morphostructural architectures of photocatalytic materials, including variations in dimensionality, surface topology, and structural hierarchy is shown in Fig. 4.

**Fig. 4:**
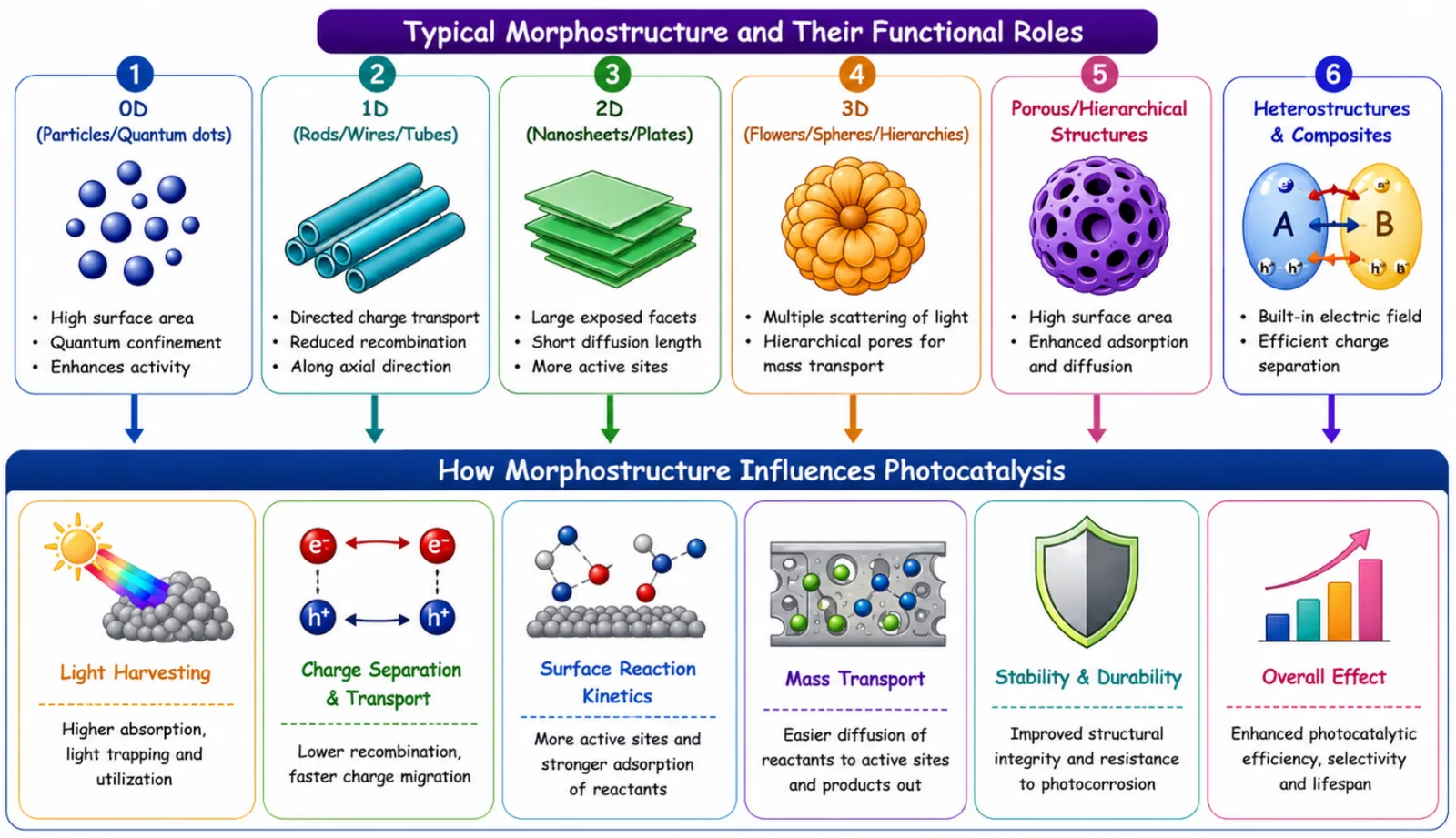
Overview of different morphostructures of photocatalytic materials across different dimensional and architectural configurations.

### 5.1 Zero-dimensional nanostructures (quantum dots and nanospheres)

Zero-dimensional nanostructures, particularly quantum dots (QDs) and nanospheres, have emerged as important photocatalytic materials because of their quantum confinement effects, high surface-to-volume ratios, tunable band structures, and abundant active sites.^169^ Semiconductor QDs such as CdS, CdSe, ZnO, InP/ZnS, and carbon quantum dots (CQDs) have demonstrated excellent performance in photocatalytic hydrogen evolution, CO_2_ reduction, and pollutant degradation due to enhanced visible-light absorption and efficient charge separation.^170^ These nanostructures are commonly synthesized through hydrothermal, solvothermal, microwave-assisted, ultrasonication, and colloidal methods. For example, Mn^2+^-doped CdS QDs exhibited improved visible-light photocatalytic activity due to intermediate electronic states that promoted light absorption and charge transfer.^140,141^ Carbon-based QDs, including CQDs, GQDs, and CNDs, are often synthesized *via* hydrothermal carbonization of biomass or organic acids. These carbonaceous QDs, when incorporated into photocatalyst composites such as g-C_3_N_4_, significantly improve visible light absorption, charge transport, and photocurrent density. It is reported that hollow tubular g-C_3_N_4_ embedded with S-doped CQDs, synthesized *via* ultrasonic-assisted self-assembly, exhibited 66.8% higher degradation efficiency for naproxen compared to pristine g-C_3_N_4_ due to enhanced charge separation and light scattering within the hollow architecture.^171^ Nanospheres, often prepared *via* hard-template or emulsion-based methods, also constitute a class of 0D photocatalytic structures. In one study, hollow nanospheres of g-C_3_N_4_/CD hybrids with diameters around 380 nm and a shell thickness of 80 nm showed a sevenfold increase in specific surface area (42.5 m^2^ g^−1^) compared to pristine g-C_3_N_4_ (6.09 m^2^ g^−1^). This enhancement contributed to greater photon harvesting, internal reflection-induced electron–hole pair generation, and higher adsorption of pharmaceuticals such as naproxen, resulting in superior photocatalytic performance.^172^ Another literature study reported that CQDs integrated into g-C_3_N_4_*via* solvothermal treatment resulted in microsphere composites with significant improvements in methyl orange degradation under visible light due to synergistic π–π interactions and interfacial charge transfer.^173^

The main limitation of 0D nanostructures is aggregation, which can reduce accessible surface area and lower performance; therefore they are often integrated into 2D or composite platforms for better stability and recyclability.^174^ Various 0D materials, including metal chalcogenides, alloy QDs, perovskite QDs, and hybrid semiconductor composites, have shown remarkable photocatalytic activity for hydrogen production and dye degradation under visible light.^175^In general, 0D materials are best viewed as highly tunable photocatalytic building blocks rather than stand-alone catalysts, especially when optimized through bandgap engineering.

Various 0D photocatalytic nanostructures, including metal chalcogenide QDs (CdS, CdSe, and ZnS), alloy QDs (Cu–In–Zn–S), carbon quantum dots (CQDs and GQDs), perovskite QDs, and hybrid semiconductor composites, have demonstrated remarkable photocatalytic performance. A few notable examples include Cu–In–Zn–S QDs yielding 456.4 µmol g^−1^ h^−1^ of H_2_ under visible light,^176^ Cd_1−*x*_Zn_*x*_Se producing 5.0 mmol g^−1^ over 6 h,^177^ and AuCu/CaIn_2_S_4_ demonstrating 452.8 µmol g^−1^ h^−1^ hydrogen evolution.^178^ Furthermore, ZnS–CuInS_2_ composites achieved 100% degradation of rhodamine B dye within 2 hours under visible light,^179^ reflecting the strong redox potential and high photon utilization of 0D nanostructures. Carbon-dot-based photocatalysts further improve visible-light absorption, interfacial charge transfer, and antibacterial activity. Owing to their tunable optoelectronic properties, high surface reactivity, and ease of heterojunction formation, 0D nanostructures remain highly promising for solar fuel generation and environmental remediation.^180^ Future research is expected to focus on scalable, low-toxicity, and multifunctional 0D photocatalysts with optimized band structures and improved charge dynamics to meet the growing demands of sustainable energy and environmental remediation.

### 5.2 One-dimensional nanostructures (nanorods, nanowires, nanotubes, and nanofibers)

One-dimensional nanostructures such as nanorods, nanowires, nanotubes, and nanofibers are important photocatalysts because their high aspect ratio promotes directional charge transport, shortens the diffusion length, and reduces electron–hole recombination.^181^ Their extended surface area also increases active-site exposure and improves light harvesting, which is why they are widely used in pollutant degradation, H_2_ evolution, CO_2_ reduction, and selective organic transformations.^181,182^ The hydrothermal and solvothermal methods are particularly prominent due to their simplicity, lower cost, and ability to yield crystalline nanostructures with controlled dimensions. Traditionally g-C_3_N_4_ nanorods have been synthesized using hard-template approaches;^183^ however, recent work by Zhu *et al.* demonstrated a template-free, self-assembly route to obtain protonated g-C_3_N_4_ nanorods.^184^ This method involves protonation and exfoliation of bulk g-C_3_N_4_, followed by curling of the nanosheets into 1D rods. When these nanorods were further functionalized with negatively charged CQDs, the resulting heterojunction system exhibited a hydrogen evolution rate of 382 µmol h^−1^ g^−1^ under visible light, owing to enhanced light harvesting, improved charge transport, and stronger interfacial interactions. Also, nanorod composites of g-C_3_N_4_ and graphene quantum dots synthesized *via* a one-step hydrothermal treatment revealed a 3.46-fold higher photocatalytic degradation rate of antibiotics compared to bulk g-C_3_N_4_, further confirming the utility of such 1D morphologies.^185^

Nanotubes, an important subclass of 1D photocatalytic materials, possess hollow geometries that enhance internal light scattering, photon multireflection, and reactant diffusion, thereby improving quantum efficiency and photocatalytic activity. CQD-embedded g-C_3_N_4_ nanotubes synthesized *via* thermal condensation of urea and carbon-dot precursors exhibited enhanced photocurrent response and hydrogen evolution owing to strong interfacial amide bonding and efficient heterojunction-mediated charge separation.^150^ Similarly, hollow tubular g-C_3_N_4_ prepared from melamine–cyanuric acid supramolecular assemblies and modified with S-doped CQDs demonstrated superior antibiotic degradation and bacterial disinfection performance due to improved visible-light absorption and shortened charge-transfer pathways.^151^

Other 1D architectures, including nanowires and nanofibers, have also demonstrated excellent photocatalytic performance. Vertically aligned ZnO nanowires synthesized by low-temperature chemical vapor deposition exhibited large diameter-to-height ratios and provided nearly 100 times higher effective surface area than flat substrates, resulting in enhanced photon harvesting and shorter charge diffusion pathways for efficient photocatalysis. Similarly, carbon nanotubes (CNTs) possess high electrical conductivity, chemical stability, and mechanical strength, making them effective supports for hybrid photocatalysts such as TiO_2_/CNT composites. These systems exhibit improved pollutant degradation and hydrogen evolution performance owing to enhanced electron transport and interfacial charge transfer.^152–155^

Although the fabrication of uniform and well-aligned 1D photocatalysts requires precise control over synthesis conditions and these structures may suffer from long-term instability under irradiation, many of these limitations have been overcome through heterostructure engineering and dopant-assisted stabilization strategies. A wide variety of 1D materials, including TiO_2_ nanorods and nanotubes, ZnO nanowires, WO_3_ and BiVO_4_ nanofibers, g-C_3_N_4_ nanotubes, and CNT-based composites, have demonstrated excellent performance in solar energy conversion, CO_2_ reduction, pollutant degradation, dye removal, and environmental remediation.^181^ Advanced fabrication techniques such as hydrothermal, solvothermal, template-free self-assembly, and chemical vapor deposition have further enhanced their structural stability, charge transport, and photocatalytic efficiency.^186^

### 5.3 Two-dimensional nanostructures (nanosheets, thin films, and layered structures)

Two-dimensional nanostructures, including nanosheets, ultrathin films, and layered frameworks, have attracted tremendous attention in photocatalysis because of their ultrathin thickness, large lateral dimensions, high specific surface area, and abundant exposed active sites.^187,188^ These unique structural features facilitate rapid migration of photogenerated charge carriers, shorten diffusion pathways, and suppress electron–hole recombination, thereby significantly enhancing photocatalytic efficiency.^189,190^ Furthermore, 2D materials provide excellent platforms for constructing heterojunctions and Z- & S-scheme photocatalytic systems with other semiconductors, enabling improved interfacial charge transfer and stronger redox capability through effective band alignment engineering.^191^ Various synthesis strategies including liquid-phase exfoliation, CVD, hydrothermal and solvothermal methods, and soft-template-assisted approaches have been extensively employed for fabricating 2D photocatalysts with tailored morphology and electronic properties.^187^ Among emerging 2D photocatalytic materials, MXenes such as Ti_3_C_2_T_*x*_ have gained particular interest because of their metallic conductivity, hydrophilic surface terminations, tunable surface chemistry, and excellent electron transport properties.^190^ Typically, Ti_3_C_2_T_*x*_ MXene is synthesized by selectively etching the Al layers from Ti_3_AlC_2_ MAX phases followed by delamination to obtain single- or few-layer nanosheets. These ultrathin MXene sheets serve as efficient cocatalysts or electron mediators in photocatalytic composites such as CdS/Ti_3_C_2_ and TiO_2_/Ti_3_C_2_, where they facilitate rapid charge separation and suppress charge carrier recombination.^192^ The strong interfacial contact between MXenes and semiconductor photocatalysts also improves visible-light absorption, enhances surface reaction kinetics, and promotes photocatalytic hydrogen evolution and organic pollutant degradation.

One study reported that a Ni_2_P/TiO_2_/Ti_3_C_2_ ternary composite achieved an impressive hydrogen evolution rate of 9425 ppm g^−1^ h^−1^ due to efficient interfacial charge transport and abundant reaction sites on the 2D MXene support.^193^ Also, g-C_3_N_4_ nanosheets prepared through thermal oxidation etching method, exhibit higher activity for visible-light-driven degradation of dyes and antibiotics, particularly when coupled with quantum dots or doped with heteroatoms like sulfur or thermal polycondensation of urea or melamine oxygen.^194^ Thin film-based 2D photocatalysts, such as TiO_2_ or ZnO deposited onto substrates by ALD or dip-coating methods, also show enhanced durability, recyclability, and interfacial contact in photocatalytic systems. It is reported that TiO_2_ thin films modified with 1 wt% graphene by a sol–gel route exhibited nearly double the efficiency for volatile organic compound (VOC) degradation and NO_*x*_ abatement under solar light compared to pristine TiO_2_. This enhancement was attributed to improved electron mobility and delayed charge recombination facilitated by the 2D graphene layer.^195^ Zheng *et al.* studied that a representative 2D-Co_2_TCPE MOF synthesized for CO_2_ reduction exhibited a remarkable CO production rate of ≈2560 µmol g^−1^ h^−1^ under visible light, outperforming its Ni-based analogue due to superior charge separation and surface exposure.^196^

A wide range of materials with 2D morphologies have been employed in photocatalysis. These include g-C_3_N_4_, GO, rGO, MXenes (Ti_3_C_2_ and V_2_C), transition metal dichalcogenides (MoS_2_ and WS_2_), LDHs, and MOFs like PCN-601 and NH_2_-MIL-125(Ti).^197^ Reported photocatalytic performances include a g-C_3_N_4_/TiO_2_ composite showing visible-light degradation of methylene blue with efficiency over 90%, a Ti_3_C_2_/g-C_3_N_4_ hybrid for CO_2_ photoreduction demonstrating superior selectivity and conversion rates, and a 2D MOF-based system achieving hydrogen evolution rates above 200 µmol g^−1^ h^−1^ with over 75% selectivity.^198^ These photocatalysts face challenges related to aggregation during synthesis or storage, limiting their active surface area. To produce defect-free ultrathin layers at large scale remains difficult and, in few cases, they may suffer from limited light absorption or poor conductivity if not engineered properly. Strategies such as doping, hybridization with conductive supports (CNTs and graphene), and hierarchical assembly into 3D architectures are employed to overcome these limitations and enhance the structural robustness of 2D materials.

### 5.4 Three-dimensional nanostructures (porous, hierarchical, flower-like, and hollow)

Three-dimensional nanostructured photocatalysts, including porous, hierarchical, flower-like, hollow, and interconnected architectures, have attracted considerable attention because of their superior structural and photocatalytic properties. The inherent advantages of 3D morphologies arise from their ability to maximize exposure of catalytically active sites, facilitate rapid mass transport, and enhance multiple light scattering and photon harvesting within the structure, thereby significantly improving photocatalytic efficiency.^199^ Furthermore, the interconnected porous networks and tunable pore structures of 3D photocatalysts provide abundant reaction interfaces and shortened diffusion pathways for photogenerated charge carriers, which effectively suppress electron–hole recombination and improve charge separation efficiency. As a result, these architectures have demonstrated exceptional performance in dye degradation, CO_2_ photoreduction, photocatalytic hydrogen evolution, bacterial disinfection, and environmental remediation applications. The fabrication of 3D photocatalysts generally involves several advanced synthetic approaches including hard-template and soft-template methods, hydrothermal and solvothermal synthesis, spray drying, electrospinning, and self-assembly techniques. Among these methods, template-assisted synthesis remains one of the most widely employed strategies for constructing hollow, porous, and hierarchical architectures with precisely controlled morphology and pore distribution. Hard templates such as silica spheres, carbon materials, and polymer beads are commonly used to fabricate hollow and ordered porous structures, whereas soft templates including surfactants, micelles, and emulsions enable the formation of flexible mesoporous frameworks.^199^ These synthetic approaches facilitate the production of photocatalysts with high surface area, improved reactant adsorption, enhanced light utilization, and efficient charge transport properties. Consequently, 3D nanostructured photocatalysts based on TiO_2_, ZnO, g-C_3_N_4_, BiVO_4_, and MOFs have exhibited significantly enhanced photocatalytic activity compared with their bulk or lower-dimensional counterparts.^90^

A study revealed that porous g-C_3_N_4_ was synthesized using SiO_2_ as a hard template followed by selective etching with NH_4_HF_2_. The resulting g-C_3_N_4_/GQD composite exhibited a specific surface area of 158.07 m^2^ g^−1^ substantially higher than that of bulk g-C_3_N_4_ and achieved enhanced degradation of RhB under visible light owing to superior pollutant adsorption and broadened light absorption from GQD integration.^200^ To overcome issues associated with hazardous template removal, Liu's group developed a template-free strategy for oxygen-doped ultrathin porous g-C_3_N_4_ using CQDs, which achieved an 18.5 – fold higher degradation rate for pharmaceutical pollutants compared to pristine g-C_3_N_4_.^201^ Hierarchical and hollow structures characterized by multi-level organization and internal cavities are also highly effective for photocatalytic applications. Rong *et al.* synthesized nest-like WO_3_ hollow structure which exhibited superior visible-light-driven photocatalytic activity, thus enabling high degradation efficiency for organic dyes.^202^ In addition, 3D MOFs (UiO-66, MIL-101, and ZIF-8) and COFs with ordered porous networks provide well-defined micro and mesopores. These structures offer abundant channels for guest molecule diffusion and enable rapid interfacial charge transfer. In one study, a 3D Ni/Co-MOF composite anchored on cellulose-derived carbon aerogels demonstrated high removal efficiency (≈80%) for tetracycline and a remarkable adsorption capacity of 624.87 mg g^−1^, due to the aerogel's hierarchical porous framework.^203^ Despite their excellent performance, 3D photocatalysts still face challenges related to corrosive template removal, structural collapse, and poor scalability. Recent advances in green templating and controlled self-assembly using biodegradable templates such as calcium carbonate or glucose have enabled the fabrication of uniform hollow structures with reduced environmental impact. Furthermore, coupling 3D architectures with 2D materials or plasmonic nanoparticles (Ag and Au) improves interfacial charge separation and extends visible-to near-infrared-light photocatalytic activity.

A broad spectrum of materials with 3D architectures have been successfully used in pollutant removal, solar-driven hydrogen generation, and CO_2_ photoreduction. These include flower-like ZnO, porous TiO_2_ spheres, hollow BiVO_4_, nest-like WO_3_, 3D MOFs (UiO-66-NH_2_, and MIL-125(Ti)), and COFs. A study reported that porous g-C_3_N_4_/CQDs exhibited a 10 times improvement in tetracycline degradation compared to bulk g-C_3_N_4_,^204^ while MOF-derived TiO_2_/CuO hollow spheres achieved 95% RhB removal within 60 minutes under visible light. Another study includes 3D MIL-101(Fe)/graphene aerogels which demonstrated a CO_2_ reduction rate of over 80 µmol g^−1^ h^−1^ due to enhanced charge mobility and CO_2_ adsorption within the porous matrix. 3D photocatalysts offer remarkable advantages in terms of structural hierarchy, active surface accessibility, and internal photon management.^205^ Through judicious design of their porosity, composition, and interface engineering, these architectures can overcome intrinsic limitations of bulk and lower-dimensional materials.

#### 5.4.1 Metamaterial structures

Recently, metamaterial-based photocatalytic systems have emerged as an advanced platform for enhancing light–matter interactions and improving photocatalytic efficiency. Metamaterial structures are artificially engineered arrangements designed to produce electromagnetic, acoustic, optical, or mechanical responses that are not found in naturally occurring materials, such as a negative refractive index, localized electromagnetic field enhancement, near-perfect light absorption, and tunable plasmonic resonance.^206^ When tailored for photocatalysis “metacatalysis”, these architectures can enhance light harvesting, modify local fields, and direct charge carriers toward specific reaction sites, thereby boosting the efficiency and selectivity of photochemical transformations which makes metamaterials highly promising for environmental remediation, solar fuel generation, CO_2_ reduction, and antibacterial applications.^207^

In photocatalytic systems, metamaterials are generally integrated with semiconductors, plasmonic nanoparticles, or 2D materials to maximize visible-light utilization and interfacial charge transfer. Plasmonic metamaterials based on Au, Ag, Al, and Cu nanostructures can generate intense localized electromagnetic fields through surface plasmon resonance, thereby increasing photon absorption and accelerating electron–hole separation.^47^ Metamaterial-assisted TiO_2_, ZnO, WO_3_, and g-C_3_N_4_ photocatalysts have demonstrated significantly improved photocatalytic degradation and hydrogen evolution because of enhanced optical confinement and prolonged light interaction times.^208^ In addition, metasurface architectures and hyperbolic metamaterials provide efficient pathways for directional charge transport and photothermal enhancement, which further improve catalytic kinetics under visible and near-infrared irradiation.^209^ Recent studies have also demonstrated that combining metamaterials with heterostructures, defect engineering, MXenes, graphene, and metal–organic frameworks (MOFs) can synergistically improve photocatalytic performance by simultaneously enhancing light harvesting, carrier mobility, and surface reaction activity.^210^ Plasmonic metamaterial-semiconductor hybrids have shown superior dye degradation and water-splitting efficiency owing to enhanced near-field coupling and interfacial electron transfer.^211^ Similarly, metamaterial-inspired hierarchical nanostructures exhibit improved adsorption and multiple internal light reflections, resulting in enhanced utilization of incident photons.^212^ Despite these advantages, challenges related to fabrication complexity, scalability, structural stability, and high production cost still limit large-scale applications. Nevertheless, ongoing advances in nanofabrication, self-assembly, nanoimprinting, and low-cost plasmonic materials are expected to accelerate the development of metamaterial-assisted photocatalysts for next-generation sustainable energy and environmental technologies.^213^

### 5.5 Core–shell structures

Core–shell structured photocatalysts consist of one component (core) encapsulated by another material (shell), forming a unique heterostructured architecture capable of significantly enhancing photocatalytic performance.^214^ This configuration plays a crucial role in improving charge separation, promoting directional charge transfer, inducing surface plasmon resonance effects, and passivating surface defects, thereby reducing electron–hole recombination losses.^215^ In most systems, the core functions as the primary light absorber or electron donor, while the shell layer facilitates charge extraction, suppresses photocorrosion, improves chemical stability, and provides additional active reaction sites. The strong interfacial interaction and synergistic coupling between the core and shell components make these architectures highly effective for solar water splitting, CO_2_ photoreduction, organic pollutant degradation, antibacterial applications, and visible-light-driven photocatalysis.^215^

A broad range of synthetic approaches has been employed for fabricating core–shell photocatalysts, including sol–gel coating, hydrothermal and solvothermal synthesis, chemical deposition, ALD, electrochemical methods, and photoinduced reduction techniques.^215^ These methods enable precise control over shell thickness, interface quality, morphology, and composition, which are critical factors governing photocatalytic efficiency. Semiconductor–semiconductor, metal–semiconductor, and dielectric-semiconductor core–shell systems such as TiO_2_@SiO_2_, Au@TiO_2_, CdS@ZnS, Fe_3_O_4_@TiO_2_, and g-C_3_N_4_@ZnO have demonstrated remarkable photocatalytic performance due to enhanced light harvesting, improved charge carrier mobility, and superior structural stability.^216^ Particularly, plasmonic metal cores such as Au and Ag can induce strong localized surface plasmon resonance, thereby extending visible-light absorption and accelerating interfacial electron transfer processes.^90^ Furthermore, shell layers can effectively protect sensitive semiconductor cores from photocorrosion while simultaneously improving adsorption capacity and surface catalytic activity.^212^

Gao *et al.* reported a photoinduced deposition method to prepare Ag@Ni core–shell nanoparticles supported on TiO_2_, where Ag acted as an electron collector and Ni as a low-cost interfacial active site.^217^ This configuration enhanced H_2_ generation by 5.4 – fold compared to conventional Ag/TiO_2_ composites, attributed to effective charge separation and interfacial catalytic activity. Another reported study includes the fabrication of an Fe_3_O_4_@COF–Au core–shell composite through stepwise chemical synthesis, which fully degraded methylene blue dye within 90 minutes under visible light. The excellent activity was credited to the integrated functionalities of COF shells, which enabled charge transfer mediation, and magnetic Fe_3_O_4_ cores for easy recovery.^218^ Moreover, hydrothermal routes followed by calcination were used to synthesize Au@CeO_2_, a plasmonic core–shell photocatalyst. This structure exhibited superior H_2_ generation (16.2 µmol mg^−1^) with an apparent quantum efficiency of 19.46% at 550 nm, owing to electromagnetic radiation effects, hot electron transfer, and plasmon resonance energy transfer across the Au–CeO_2_ interface.^219^ The advantages of core–shell photocatalytic morphologies are multifaceted and strongly associated with their ability to regulate charge transfer and interfacial interactions at the nanoscale. One of the major benefits of these architectures is the significant reduction in electron–hole recombination through efficient spatial separation of photogenerated charge carriers across the core–shell interface, which substantially enhances photocatalytic efficiency. In many systems, the shell layer acts as a protective barrier that suppresses photocorrosion and prevents direct contact of sensitive core materials with reactive species, thereby improving long-term stability, durability, and recyclability of the photocatalyst.^46^ In plasmonic core–shell structures, metallic cores such as Au or Ag can induce localized surface plasmon resonance, while the shell modulates the local electromagnetic field and facilitates enhanced light harvesting and interfacial electron transfer. Furthermore, carefully engineered band alignment at the core–shell interface enables the formation of efficient Type-II heterojunctions or Z-scheme charge transfer pathways, thereby improving redox capability and photocatalytic performance.

Several studies have shown the effectiveness of such architectures in environmental and energy-related applications. In one study, MnO_2_/TiO_2_ core–shell nanostructures synthesized by a microwave-assisted hydrothermal method showed highly efficient visible-light-driven removal of gaseous mercury and organic pollutants.^220^ The enhanced performance was mainly attributed to improved interfacial charge transfer and favorable band structure alignment. Similarly, Au@TiO_2_, CdS@ZnS, and Fe_3_O_4_@TiO_2_ systems have shown enhanced photocatalytic hydrogen evolution, dye degradation, and antibacterial activity owing to synergistic interactions between the core and shell components.^221^ Despite these advantages, fabrication of core–shell photocatalysts still presents several challenges. Precise control over shell thickness, morphology, and interface composition is essential because excessive shell coverage may hinder light absorption and block active catalytic sites.^215^ Additionally, lattice mismatch between core and shell materials can induce interfacial strain and defects that negatively affect charge transport and photocatalytic efficiency.^46^ Scalability and reproducibility remain important concerns, particularly in noble-metal-based systems involving Au and Ag. Nevertheless, advances in interface engineering and controlled deposition techniques continue to improve the practicality and performance of core–shell photocatalysts for environmental and energy-related applications.^222^

Numerous materials like metal–metal oxide combinations such as Ag@TiO_2_, Au@CeO_2_, Ag@Ni/TiO_2_; semiconductor–semiconductor systems like WO_3_@TiO_2_ and ZnO@SnO_2_; and hybrid organic-inorganic materials such as Fe_3_O_4_@COF–Au and MOF@COF composites have been employed in core–shell photocatalyst designs.^212,223^ Chen *et al.* reported MOF-based core–shell structures such as NH_2_-UiO-66@COF which exhibited a H_2_ evolution rate of 7178 µmol g^−1^ h^−1^ under visible light, far outperforming individual MOF or COF components.^224^ Another report demonstrated a core–shell MOF composite achieving 5.06 mmol h^−1^ g^−1^ NADH photocatalytic conversion efficiency by promoting electron–hole separation across the MOF-COF interface.^225^ The core–shell structured photocatalysts can be utilized in environmental remediation and sustainable energy generation.

### 5.6 Heterostructures (Z-scheme, p–n junctions, and lateral/vertical)

Heterostructured photocatalysts formed by coupling two or more semiconductors with different band structures have emerged as one of the most effective strategies for improving photocatalytic hydrogen evolution, CO_2_ reduction, pollutant degradation, and antibacterial activity.^226^ Their key advantage lies in suppressing rapid recombination of photogenerated electron–hole pairs through efficient interfacial charge separation and directional carrier transfer. Depending on band alignment, heterojunctions are classified as type-I, type-II, Z-scheme, and S-scheme, each balancing charge transfer efficiency and redox capability differently. Z-scheme systems mimic natural photosynthesis by recombining less useful carriers while preserving strong redox potentials: in a direct Z-scheme, electrons from the conduction band (CB) of the Oxidation Photocatalyst (OP) recombine with holes in the valence band (VB) of the Reduction Photocatalyst (RP), leaving high-energy electrons in RP-CB and holes in OP-VB, enabled by mediators or direct contact.^227^ S-scheme heterojunctions refine this by creating staggered band alignment between RP and OP with Fermi-level differences, forming built-in electric fields that drive recombination of electrons in OP-CB and holes in RP-VB, while retaining highly reactive carriers and eliminating the need for external mediators, thus simplifying fabrication and improving stability.^228^

The fundamental advantage of heterostructures is enhanced charge separation and directional carrier migration across engineered interfaces, significantly boosting photocatalytic activity in water splitting, CO_2_ reduction, pollutant degradation, and antibacterial applications.^90^ Representative systems such as g-C_3_N_4_/TiO_2_, CdS/NiI_2_, WO_3_/SnS_2_, g-C_3_N_4_/graphene composites, and Ag/Ag_3_PO_4_/WO_3_ show improved visible-light performance due to better charge separation, plasmonic effects, and multiple junctions.^229,230^ Lateral and vertical heterostructures of g-C_3_N_4_ with carbon dots exhibit excellent hydrogen evolution, with lateral covalent configurations reaching up to 24 760 µmol h^−1^ g^−1^, far exceeding the single-component system.^231^ Z-scheme heterostructures provide the dual benefits of strong redox potential and efficient charge transfer by allowing selective recombination of minority carriers at the junction interface. This mechanism has been further optimized in all-solid-state Z-schemes using conductive mediators like graphene or metallic nanoparticles to facilitate electron migration. It is noted that a ternary g-C_3_N_4_/BiVO_4_/Ag system exhibited a 5-fold enhancement in visible-light-driven water splitting due to synergistic band matching and SPR effects.^232^ Also, S-scheme heterojunctions like ZnO/CdS coupled with GO have demonstrated improved photocatalytic disinfection and dye degradation due to enhanced interfacial polarization and directional carrier migration.^233^

Heterostructures also face challenges including maintaining intimate and stable interfaces, avoiding parasitic recombination, and achieving scalable, uniform synthesis; mediator-based Z-schemes are particularly prone to degradation and efficiency loss over time. All-solid-state Z-scheme and S-scheme architectures using graphene or metallic nanoparticles mitigate these issues by relying on intrinsic band alignment and built-in fields. Two-dimensional materials (MXenes, graphene, and LDHs) and porous frameworks (MOFs and COFs) further extend the design space for hierarchical, tunable photocatalysts.^234^ A wide range of heterostructures such as TiO_2_/ZnO, WO_3_/g-C_3_N_4_, Bi_2_O_3_/Ta_3_N_5_, Ti_3_C_2_@TiO_2_/CuInS_2_, ZnFe_2_O_4_/WO_3_, NaNbO_3_/WO_3_, and TiO_2_/WO_3_/TiO_2_ double heterojunctions, have demonstrated strongly enhanced hydrogen evolution and dye-pollutant degradation under solar and visible light.^192,235^ Also, TiO_2_/WO_3_/TiO_2_ double heterojunctions demonstrated enhanced methyl orange degradation under near-UV and visible light, attributable to the layered junction architecture and reduced charge recombination. These heterostructures are found to be useful in solar energy conversion and pollutant degradation.^236,237^ Continuous advances in synthesis, interfacial engineering, and multicomponent design are therefore expected to accelerate the practical deployment of heterostructured photocatalysts in sustainable energy conversion and environmental remediation.

### 5.7 Defect-engineered structures

Defect-engineered photocatalysts have rapidly gained recognition as a transformative class of semiconducting materials due to their ability to modulate intrinsic electronic, optical, geometrical and chemical properties through the intentional introduction of atomic-scale irregularities.^238^ These materials utilize structural defects such as oxygen vacancies, lattice distortions, surface vacancies, dopant-induced disorders, interstitial defects, and heteroatom substitutions to modify the electronic band structure and create intermediate energy states within the band gap.^90^ The presence of such defect states can significantly enhance visible-light absorption, improve carrier mobility, and facilitate charge separation by trapping photogenerated electrons or holes, thereby prolonging charge carrier lifetimes and suppressing recombination losses.^239^ This strategy has proven particularly effective for materials such as WO_3_, TiO_2_, ZnO, and g-C_3_N_4_, where intrinsic limitations including wide band gaps and rapid charge carrier recombination can be overcome through controlled defect engineering. Chen *et al.* demonstrated that defect-induced disorder and oxygen vacancies in TiO_2_ significantly enhanced visible-light absorption and charge separation.^43^ Similarly, Pan *et al.* reported that oxygen-vacancy engineering effectively improves photocatalytic performance by promoting carrier transport and extending light absorption into the visible region.^240^ The synthesis of defect-rich photocatalysts can be achieved through a variety of chemical and physical routes.In addition, defect sites frequently act as catalytically active centers that promote adsorption and activation of reactant molecules, ultimately enhancing photocatalytic redox reactions.^241^ Several studies have further shown the effectiveness of defect engineering in improving photocatalytic performance by improving light absorption, charge separation, and surface reactivity. Defect-rich WO_3_ nanosheets exhibited a 2.5-fold increase in rhodamine B degradation and a 1.7-fold enhancement in hydrogen evolution compared with pristine WO_3_. Similarly, oxygen-deficient WO_3_−x/g-C_3_N_4_ Z-scheme heterostructures achieved a CO_2_ reduction rate of 155 µmol g^−1^ h^−1^, significantly outperforming non-defective systems.^242^ In another study, a defect-engineered g-C_3_N_4_/graphene composite showed a 3.4-fold improvement in visible-light-driven hydrogen evolution due to enhanced light absorption and efficient interfacial charge separation. Coupling vacancy defects with advanced architectures such as nanosheets and hierarchical hollow spheres further enhances photocatalytic efficiency by increasing exposure of active defect sites and improving adsorption and mass transport.

Among the most common methods are controlled thermal treatments in reductive atmospheres, hydrothermal/solvothermal synthesis with defect-inducing agents, and doping with heteroatoms (N, C, S, and B). For example, oxygen vacancies in WO_3_ nanosheets were successfully introduced *via* lower temperature reduction processes, resulting in improved visible light absorption and enhanced photocatalytic reduction of oxidized uranium U(vi)^243^ and degradation of gaseous toluene.^244^ Moreover, defect-rich g-C_3_N_4_ prepared by protonation and ultrasonication techniques exhibited increased specific surface area, visible light response, and active site density.^245–247^ In addition, Pt-decorated WO_3_ nanosheets with engineered oxygen vacancies exhibited improved separation and collection of photogenerated charges, resulting in superior oxidative decomposition of VOCs.^248^ Furthermore, polymeric carbon nitride engineered with nitrogen and carbon vacancies showed enhanced CO_2_ photoreduction efficiency due to synergistic charge delocalization and interface polarization. Despite its advantages, defect engineering requires precise control because excessive defects can create deep trap states, promote non-radiative recombination, reduce crystallinity, and weaken structural stability or photocorrosion resistance. Recent approaches such as atomic layer etching, plasma-assisted doping, and co-catalyst-mediated defect passivation have enabled better regulation of defect density and distribution, thereby balancing photocatalytic activity and stability. Shoueir *et al.* reported that noble-metal-assisted photodeposition in Au/TiO_2_ systems reduced Ti^4+^ to Ti^3+^, introducing shallow trap states and extending visible-light absorption while preserving lattice stability.^249^ Similarly, carbon doping in ZnO and TiO_2_ generated surface and bulk defects that narrowed the band gap from 3.2 to ∼2.5 eV, thereby enhancing sunlight-driven photocatalytic activity.

Table 3 below categorizes various photocatalyst morphologies reported in the literature, along with representative materials and their primary photocatalytic applications.

**Table 3:** Classification of photocatalyst-based morphostructure and respective applications

Morphostructure type	Representative materials	Synthesis techniques	Photocatalytic applications	Advantages	Ref.
0D (quantum dots and nanospheres)	CdSe@ZnS QDs, ZnO QDs, and InP/ZnS QDs	Green synthesis, hydrothermal, and microwave	Water splitting, dye degradation, and CO_2_ reduction	High surface energy, quantum confinement, and excellent visible light absorption	169
1D (nanorods, nanotubes, nanowires, and nanofibers)	TiO_2_ NRs, WO_3_ NRs, and ZnO nanorods	Solvothermal, hydrothermal, and electrospinning	Organic pollutant degradation and photoelectrochemical H_2_ generation	Enhanced electron transport, reduced recombination, and large surface-to-volume ratio	181
2D (nanosheets, thin films, and layered structures)	g-C_3_N_4_ sheets, BiVO_4_, and TiO_2_ nanosheets	Sonochemical, sol–gel, microwave, and exfoliation	Antibiotic degradation, solar fuel production, and air purification	High anisotropic charge mobility, light scattering effect, and increased active surface area	187
3D (porous, hierarchical, flower-like, and hollow)	ZnO microspheres, MOFs (ZIF-8 and UiO-66), and Bi_2_O_3_	Sol–gel, solvothermal, templating, and microwave	Dye degradation, hydrogen evolution, and CO_2_ conversion	Enhanced mass transfer, multiple light scattering, and more reaction sites	199
Core–shell structures	Ag@TiO_2_, Fe_2_O_3_@C, and TiO_2_@Ag_2_CO_3_	Co-precipitation and chemical deposition	Visible-light photocatalysis and dye degradation	Suppressed recombination, plasmonic enhancement, and interfacial charge migration	214
Heterostructured (Z-scheme, p-n junctions, and lateral/vertical)	WO_3_/g-C_3_N_4_, TiO_2_/BiVO_4_, and g-C_3_N_4_/CDs composites	Solvothermal, ALD, ultrasonic, and green synthesis	CO_2_ reduction, photocatalytic disinfection, and dual-functional catalysts	Improved charge separation, interfacial synergy, and widened light absorption spectrum	226
Defect-engineered structures	WO_3−*x*_, TiO_2_-δ, and doped g-C_3_N_4_	Thermal treatment, doping, and mechanochemical	Pollutant degradation, VOC oxidation, and hydrogen evolution	Creation of oxygen vacancies and mid-gap states improves light absorption and reactivity	238

## 6 Future directions and challenges

Some important future prospects and challenges recommended are mentioned below. These recommendations mainly focus on the relationship between the synthesis strategy, morphostructural control, and charge-transfer mechanism, to enhance the photocatalytic performance. These also highlight the economic feasibility in future photocatalyst development.

(i) Future photocatalyst development should focus on synthesis-controlled structural engineering rather than only improving apparent photocatalytic activity. Greater emphasis is needed for understanding how synthesis conditions influence crystallinity, defect density, interface formation, and charge-carrier dynamics.

(ii) Advanced synthesis methods such as atomic layer deposition, microwave-assisted synthesis, plasma treatment, electrospinning, and template-assisted growth should be further optimized for precise control of morphostructure and heterointerface quality. However, these methods must also be evaluated for scalability, cost, reproducibility, and industrial feasibility.

(iii) Morphostructure engineering remains a major direction for improving photocatalytic performance. Future work should systematically compare 0D, 1D, 2D, and 3D hierarchical structures under similar experimental conditions to identify the true contribution of morphology toward light harvesting, charge transport, and surface reaction kinetics.

(iv) Defect engineering should move toward controlled and measurable defect modulation instead of simply increasing vacancy concentration. Advanced characterization methods such as electron paramagnetic resonance, transient absorption spectroscopy, and *operando* analysis should be used to determine whether defects enhance charge separation or act as recombination centers.

(v) Z- & S-scheme heterojunctions continue to show strong potential for improving redox capability and charge separation. However, future studies should verify charge-transfer pathways using direct experimental measurements rather than relying mainly on band alignment diagrams.

(vi) Emerging morphostructural platforms such as MXene-based systems, quantum-dot heterostructures, metamaterials, bio-derived composites, and single-atom photocatalysts offer new opportunities for enhanced visible-light absorption and interfacial charge transfer. Their long-term stability, toxicity, synthesis reproducibility, and practical durability should be investigated more systematically.

(vii) New techniques such as artificial intelligence, computational modelling, and data-driven materials screening may help accelerate the design of photocatalysts with optimized morphostructure, controlled defects, and efficient charge-transfer pathways.

(viii) The economic feasibility of photocatalytic materials should also become a major consideration in future research. Higher performance photocatalysts prepared using expensive precursors or energy-intensive synthesis routes may face limitations in large-scale implementation. Therefore, future efforts should prioritize lower-cost, scalable, energy-efficient, and environmentally sustainable synthesis strategies while maintaining long-term photocatalytic stability and performance.

## 7 Conclusions

This review article emphasized photocatalytic performance which depends strongly on the synthesis method, morphology, defect chemistry, and interfacial charge transfer. This review shows that photocatalytic activity cannot be explained only by surface area or material composition. Instead, controlled synthesis plays a key role in determining crystallinity, defects, charge separation, and overall catalytic efficiency. Different synthesis methods provide different advantages and limitations. Conventional methods such as hydrothermal and solvothermal synthesis provide good crystallinity and morphology control, while sol–gel and co-precipitation methods offer simplicity and scalability, whereas the advanced techniques including ALD, plasma treatment, and defect engineering provide better control over defects and interfaces but may increase cost and complexity. Also, morphostructures such as nanorods, nanosheets, hierarchical structures, and Z- & S-scheme heterojunctions can improve charge transport and light absorption, but their benefits are not always universally reproducible under different experimental conditions. The review further underlines that many reported hypothetical mechanisms are without appropriate experimental support. Therefore, future studies should combine photocatalytic performance measurements with direct mechanistic validation techniques and optimization. More attention should also be given to reproducibility, long-term stability, defect optimization, scalability, and standardized testing procedures. Thus, the development of efficient and scalable photocatalytic materials requires an integrated approach that combines controlled synthesis, structural design, mechanistic understanding, and realistic performance evaluation. We assume that these strategies will help bridge the gap between laboratory studies and reliable real-world applications of photocatalytic materials in environmental remediation and solar energy conversion.

## Conflicts of interest

There are no conflicts to declare.

## Data Availability

No primary research results, software or code have been included and no new data were generated or analysed as part of this review.
